# Inorganic Agents for Enhanced Angiogenesis of Orthopedic Biomaterials

**DOI:** 10.1002/adhm.202002254

**Published:** 2021-05-26

**Authors:** Monika Šalandová, Ingmar A. J. van Hengel, Iulian Apachitei, Amir A. Zadpoor, Bram C. J. van der Eerden, Lidy E. Fratila‐Apachitei

**Affiliations:** ^1^ Additive Manufacturing Laboratory Department of Biomechanical Engineering Faculty of Mechanical, Maritime, and Materials Engineering Delft University of Technology Mekelweg 2 Delft 2628 CD The Netherlands; ^2^ Department of Internal Medicine Erasmus Medical Center Doctor Molewaterplein 40 Rotterdam 3015 GD The Netherlands

**Keywords:** angiogenesis, bone regeneration, orthopedic implants, trace elements

## Abstract

Aseptic loosening of a permanent prosthesis remains one of the most common reasons for bone implant failure. To improve the fixation between implant and bone tissue as well as enhance blood vessel formation, bioactive agents are incorporated into the surface of the biomaterial. This study reviews and compares five bioactive elements (copper, magnesium, silicon, strontium, and zinc) with respect to their effect on the angiogenic behavior of endothelial cells (ECs) when incorporated on the surface of biomaterials. Moreover, it provides an overview of the state‐of‐the‐art methodologies used for the in vitro assessment of the angiogenic properties of these elements. Two databases are searched using keywords containing ECs and copper, magnesium, silicon, strontium, and zinc. After applying the defined inclusion and exclusion criteria, 59 articles are retained for the final assessment. An overview of the angiogenic properties of five bioactive elements and the methods used for assessment of their in vitro angiogenic potential is presented. The findings show that silicon and strontium can effectively enhance osseointegration through the simultaneous promotion of both angiogenesis and osteogenesis. Therefore, their integration onto the surface of biomaterials can ultimately decrease the incidence of implant failure due to aseptic loosening.

## Graphical abstract

Aseptic loosening of a permanent prosthesis remains one of the most common reasons for bone implant failure. To improve the fixation between implant and bone, and enhance angiogenesis, bioactive agents are incorporated into the surface of biomaterials. This review compares the angiogenic potential of five bioactive elements and provides an overview of the in vitro methodologies used for their assessment.

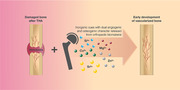

## 1 Introduction

Despite the great technological advancements in total joint replacements (TJRs) over the past decades, implant failure remains a concern for ≈10% of patients undergoing primary total hip arthroplasty.^[^ ^1^, ^2^ ^]^ Many of the causes leading to failures are attributed to poor or delayed osseointegration of the permanent implants,^[^ ^2^, ^3^ ^]^ as it has been established that achieving osseointegration is a key prerequisite for implant stability and proper loading of the implant.^[^ ^2^, ^4^, ^5^ ^]^ Unsatisfactory osseointegration is often associated with the formation of fibrous tissue between the biomaterial and the bone, which represents a soft interlayer not able to sufficiently anchor the implant. Moreover, an unsecured attachment can result in micromovements and subsequent generation of wear debris, which may elicit an inflammatory reaction and excessive bone resorption, eventually leading to the loosening of the prosthesis.^[^ ^6^, ^7^, ^8^, ^9^, ^10^ ^]^

Presently, metallic and ceramic biomaterials are used for the majority of load‐bearing orthopedic implants due to their high strength.^[^ ^11^, ^12^ ^]^ Bioinert alumina and zirconia ceramics demonstrate superiority in hardness and wear resistance among available biomaterials resulting in minimal immune response, which makes them extremely suitable for the fabrication of the articulating components of TJRs, such as femoral heads.^[^ ^10^, ^12^ ^]^ Among metallic biomaterials, titanium alloys are increasingly used for TJRs. They are often praised for their high corrosion resistance and moderate elastic modulus, the latter reducing the stress shielding effect and preventing undesired bone resorption.^[^ ^6^, ^7^, ^9^ ^]^ Even though these biomaterials exhibit an exemplary chemical and mechanical stability, their bioinert nature does not encourage the establishment of a stronger and more physiological connection between the implant and the new bone, thus necessitating further surface treatment of the implants.^[^ ^2^, ^10^, ^11^ ^]^ Many of the approaches currently used to promote osseointegration are based on the attraction of mesenchymal stem cells (MSCs) and the stimulation of their osteogenic differentiation, leading to new bone tissue formation on the implant surface. This can be achieved through the adjustment of the chemical and physical surface properties of the used biomaterial.^[^ ^2^, ^4^, ^6^ ^]^

Given the highly vascularized nature of the bone^[^ ^13^, ^14^ ^]^ and the importance of blood supply in the bone repair process,^[^ ^15^ ^]^ angiogenesis plays a crucial role and remains a major challenge in bone tissue engineering and regeneration. Furthermore, the research on the effects of inorganic elements on angiogenesis is relatively scarce when compared to the research on osteogenic agents.^[^ ^16^ ^]^ Due to their vital role, damaged blood vessels are repaired through the angiogenic process in the initial stages of bone regeneration.^[^ ^5^, ^17^, ^18^ ^]^ As blood flow is restored, the delivery of oxygen, nutrients, and molecules as well as a supply of cells to the affected site, cell signaling and waste product removal are ensured.^[^ ^19^, ^20^, ^21^ ^]^ Implants with both osteogenic and angiogenic surface biofunctionalities are, therefore, highly desirable to enhance osseointegration.^[^ ^22^ ^]^

Among the available methods used for the modulation of cellular responses by an implant, modification of the chemical composition of the biomaterials is an approach that enables the incorporation of multiple agents with different action mechanisms, thereby yielding a biomaterial with versatile surface properties. Essential and trace elements are known for their inherent role in many molecular mechanisms in the human body, and the increased understanding of their signaling and structural functions associated with bone metabolism has led to their utilization in therapeutic applications for bone (e.g., osteoporotic treatments, promoting osseointegration).^[^ ^5^, ^13^, ^14^, ^23^ ^]^ The calcium (Ca) and phosphorus (P) essential elements, which are constituting the hydroxyapatite crystals found in bone, were among the first elements with osteogenic potential and recognized suitability for orthopedic applications.^[^ ^14^, ^23^ ^]^ Nowadays, trace elements such as copper (Cu), magnesium (Mg), silicon (Si), strontium (Sr), and zinc (Zn), which may additionally enhance angiogenesis, are also incorporated into bulk biomaterials or onto their surfaces, delivering their stimulatory effect to the intended site through tunable release kinetics. They can modulate the activity of stem/progenitor cells, thereby inducing new bone and/or blood vessel formation and enhancing osseointegration.^[^ ^5^, ^13^, ^23^, ^24^, ^25^ ^]^

Due to its biodegradable nature and mechanical properties comparable to the bone, Mg is an attractive metallic biomaterial for resorbable scaffolds intended for bone regeneration.^[^ ^13^, ^26^ ^]^ The presence of Mg may favor osseointegration through the recruitment of bone marrow stromal stem cells^[^ ^13^ ^]^ and more recent research has indicated its angiogenic potential through the upregulated expression of angiogenic factors.^[^ ^14^ ^]^ Sr is used as strontium ranelate (Protelos) for treating osteoporotic patients.^[^ ^23^, ^27^ ^]^ The superiority of strontium ranelate over other osteoporotic drugs is related to its ability to decouple the various processes involved in bone remodeling by promoting osteogenesis while simultaneously suppressing bone resorption.^[^ ^28^, ^29^, ^30^ ^]^ The antimicrobial activity of Cu has been utilized in the medical field for decades.^[^ ^31^ ^]^ However, this metal is also gaining increasing recognition for its wide range of catalytic and structural functions in other biological processes,^[^ ^23^, ^32^ ^]^ such as tissue regeneration.^[^ ^33^, ^34^ ^]^ As far as orthopedic applications are concerned, Cu can not only decrease the incidence of implant‐associated infections, but it could also improve bone quality around the implant by increasing its mineral density^[^ ^32^, ^35^ ^]^ and promoting the formation of a new vascular network.^[^ ^36^ ^]^ The majority of Zn found in the human body is stored within bone,^[^ ^13^, ^23^, ^32^ ^]^ reflecting its essential involvement in bone homeostasis. Zn promotes osteogenesis by regulating the activity of osteoblasts and osteoclasts^[^ ^23^, ^27^, ^32^ ^]^ and similar to Cu, it could also be employed as an antibacterial agent.^[^ ^13^ ^]^ Si is involved in bone metabolism through both anabolic and catabolic processes, it promotes bone homeostasis, regeneration, and increases its mineral density.^[^ ^37^, ^38^ ^]^ One of the introduced osteogenic mechanisms of silicon is the promotion of collagen 1 deposition and stabilization,^[^ ^32^, ^38^ ^]^ as well as the recruitment of progenitor cells through immunomodulation of monocytes.^[^ ^37^ ^]^

This review aims to provide the reader with a state‐of‐the‐art overview on the angiogenic properties of trace elements incorporated on the surfaces of permanent orthopedic biomaterials with a focus on the in vitro assays used to evaluate the response of endothelial cells (ECs) to such biomaterials, the comparative angiogenic potential of the trace elements for bone implants, and the mechanisms underlying the observed angiogenic activity.

## 2 Methods

PubMed and Web of Science were used as the primary search databases. The search terms and strategy are summarized in **Table** **1** and Figure S, Supporting Information. First, the databases were screened for the general term ECs and the selected elements. The search terms were further specified by the addition of angiogenic components and the intended applications while the period was set to the time window between 2010 and 2020. The search from both databases yielded 465 articles. After removal of duplicates, 419 articles were individually screened. Based on the relevance of the title and abstract, 109 articles were selected and further classified with consideration to the application requirements mentioned in the motivation section above. Finally, 75 articles were included for full‐text assessment out of which 58 were included in the final comparison of the five inorganic elements.

**Table 1:** Summary of inclusion and exclusion criteria

Screening	Inclusion criteria	
	Web of science	PubMed
Initial search term:	TS = ((endothelial cells) and (angiogen*) and (magnesium or mg or copper or Cu or silicon or Si or zinc or Zn or strontium or Sr) and (implant or scaffold or material))	Endothelial cells and angiogen* and (magnesium or Mg or copper or Cu or silicon or Si or zinc or Zn or strontium or Sr) and (implant or scaffold or material)
Year:	2010–2020	
First screening:	Relevance of title and abstract; discussing effect of the ions/particles on endothelial cells/angiogenesis	
Second screening:	Bone related field of application (orthopaedic/dental)	
Full‐text screening:	Effect of one of the ions on angiogenic behavior of endothelial cells	

All included articles discussed the effects of one or more of the selected ions/nanoparticles (Cu, Mg, Si, Sr, or Zn) on ECs. The composition of the tested materials, concentrations of the potential angiogenic agent (in the form of ions or nanoparticle), and the reported effects on ECs were summarized and compared. The articles were also screened for the different assessment methods of the angiogenic properties of the agents to evaluate their widespread use whilst critically reviewing their suitability, with consideration of the reliability of the output data, costs, and other general (dis)advantages (e.g., duration, complexity level, etc.). The findings were compared and completed with the results of several review articles on in vitro angiogenic assessment methods, yielding the final overview.

## 3 Angiogenesis and Its Role in Fracture Healing

**Figure 1:**
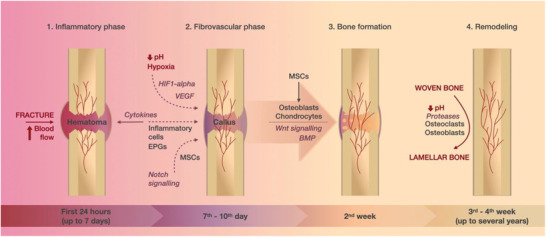
An illustration depicting the stages of fracture healing: 1) In the initial inflammatory phase (lasting up to 7 days after injury), the increased blood delivery to the affected site results in the formation of hematoma with a high content of cytokines; 2) cells attracted by cytokines and environmental factors (hypoxia, low pH, HIF1‐alpha, and VEGF) are responsible for the repair of damaged vessels and formation of provisional fibrous tissue called callus (7–10 days after injury); 3) at around two weeks after injury, MSCs undergo differentiation into osteoblasts and chondrocytes governed by Wnt and BMP signaling and provisional woven bone is generated; 4) in the final phase starting 3–4 weeks after injury and lasting up to several years, the woven bone is replaced by lamellar bone.

After a bone replacement surgery, the body suffers local tissue damage analogous to that of a fracture. The blood supply is disrupted and the local environment loses mechanical stability.^[^ ^18^, ^39^ ^]^ New bone and vascular tissue must both be generated to restore homeostasis and to secure a strong tissue‐biomaterial interface, which is vital for the success of cementless permanent implants. The mutual dependence of angiogenesis and osteogenesis has been recognized by many studies as being critical for achieving successful bone repair, as impaired angiogenic ability or significantly damaged vasculature has been associated with increased occurrence of nonunions or delayed repair.^[^ ^20^, ^21^, ^40^, ^41^, ^42^ ^]^ The fracture healing process is illustrated in **Figure** **1**.

In the immediate aftermath of tissue damage, the wound elicits inflammatory and haemostatic reactions, defined by orchestrated molecular cascades, blood vessel constriction, blood coagulation, and the formation of a fibrin‐rich blood clot at the affected site.^[^ ^20^, ^41^, ^43^ ^]^ The clot is characterized by hypoxia and low pH and serves as a temporary scaffold at the wounded site.^[^ ^20^ ^]^ It is also a source for cytokines and signaling molecules, which together with environmental factors (hypoxia) are responsible for the recruitment of MSCs, endothelial progenitor cells (EPCs), and inflammatory cells from their local sources.^[^ ^15^, ^19^, ^39^, ^43^, ^44^ ^]^

The initial inflammatory reaction has a substantial influence on the formation of a callus, that is, a fibrovascular tissue that provides a more stable support/matrix for the further development of blood vessels and bone tissue.^[^ ^18^, ^39^, ^42^ ^]^ Through reciprocal signaling, vasculature and bone mature side by side. Hypertrophic chondrocytes and cells of the osteoblastic lineage contribute to the secretion of vascular endothelial growth factor (VEGF),^[^ ^45^ ^]^ a pro‐angiogenic factor that, in synergy with several bone morphogenic proteins (BMPs), increases the recruitment of MSCs and encourages their differentiation toward osteoblasts.^[^ ^18^ ^]^ Stimulated ECs proliferate, migrate, and develop into structures to form new vessels and restore the blood flow in the callus. The vasculature surrounding and growing into the provisional fibrous tissue is vital for its replacement by the hard callus, as it enables sufficient delivery of oxygen and nutrients required for this endochondral ossification and helps to convey osterix‐positive osteoprogenitor cells from the perichondrium into the metaphysis, contributing to osteoblastogenesis inside the bone.^[^ ^15^, ^18^, ^21^, ^39^, ^40^, ^45^, ^46^, ^47^ ^]^ Finally, the provisional woven bone is remodeled through repetitive tissue resorption and deposition cycles and replaced by a functional lamellar bone.^[^ ^15^, ^39^, ^40^ ^]^

**Figure 2:**
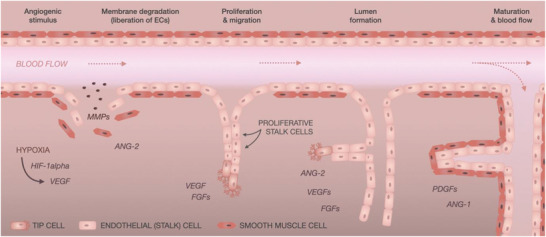
An illustration of sprouting angiogenesis. The presence of different factors (hypoxia, HIF1‐alpha, VEGF) can initiate angiogenesis, which is divided into 4 stages: 1) In the first stage, the membrane degrades resulting in the liberation of ECs; 2) the cells proliferate and migrate, thereby establishing new branches of the vascular network; 3) the new branches are initially formed without a lumen and are hollowed in a subsequent stage; 4) the new endothelium matures, and blood flow is established through the new vessels.

The vascular network can be formed via two processes, angiogenesis and vasculogenesis, which are often incorrectly interchanged despite their substantial differences. Vasculogenesis employs the EPCs, which are obtained from different sources. The recruitment of EPCs is governed by molecular (cytokines) and environmental (hypoxic) factors. These cells then further differentiate into mature ECs and develop de novo (new) blood vessels.^[^ ^17^, ^18^, ^19^, ^43^ ^]^ The importance of vasculogenesis in the onset of vascularization during embryonic development has been known for decades, but recent studies confirmed its role also postnatally.^[^ ^17^ ^]^ Angiogenesis, on the other hand, utilizes the existing vasculature and is the dominant vessel formation process in tissue repair and tumor growth. It differentiates between two mechanisms of network growth: sprouting and splitting of the blood vessels.^[^ ^17^, ^18^, ^19^, ^43^ ^]^ The latter process, also called intussusceptive angiogenesis, is usually observed in well‐perfused regions undergoing morphological changes, such as remodeling or growth. In contrast, areas with no or very little blood supply, such as wounds, are characterized by proliferative branching (sprouts) from the remaining vasculature, thus forming a new capillary network.^[^ ^17^, ^48^, ^49^ ^]^ Sprouting angiogenesis, the prevailing revascularization mechanism for fracture healing, is defined by the following stages which are also illustrated in **Figure** **2**.

1. First, the basement membrane of the blood vessels, which together with mural cells (vascular smooth muscle cells and pericytes) prevents ECs from leaving their designated location in the vascular wall, must be degraded to liberate the ECs. Major biomolecular factors of this phase include matrix metalloproteinases (MMPs), which define the extent of the membrane degradation and at the same time are responsible for the secretion of angiogenic factors, such as VEGF, fibroblast growth factor (FGF), and transforming growth factor beta, as well as activation of relevant angiogenic chemokines.^[^ ^17^, ^22^, ^50^ ^]^
2. The sprouting angiogenesis is characterized by ECs of distinct (but reversible) function and morphology. The new branches comprise of tip and stalk cells. The establishing capillaries are guided by mildly proliferative tip cells, contain many filopodia and navigate the new vessels toward a relevant (angiogenic) stimulus (hypoxia, biochemical gradient).^[^ ^17^, ^22^ ^]^
3. The new endothelial branch is initially formed as a solid cord without a lumen. The growth and branching of the new vessel are mainly determined by the proliferation of stalk cells, which, in contrast to tip cells, are characterized by fewer filopodia.^[^ ^17^ ^]^ Moreover, they are responsible for the production of the basement membrane and the establishment of junctions with neighboring cells.^[^ ^17^, ^50^ ^]^
4. Stalk cells are responsible not only for the elongation of the branches but also for lumen formation, which is achieved by the tubular arrangement of these cells. Past studies introduced two mechanisms, in which the lumen is formed either by “cell hollowing” or “cord hollowing.” The “cell hollowing” theory works on the assumption that the intracellular vacuoles of adjacent ECs connect, thus creating inner space. The more recent “cord hollowing” theory, on the other hand, explains the lumen formation with cells acquiring a distinct phenotype, subsequent rearrangement of neighboring cells, and lumen opening as a result of repulsive forces on the established inner membrane.^[^ ^17^, ^50^, ^51^ ^]^
5. Once the lumen is established, the blood flow initiates. The contiguous tubular branches are then coalesced, forming an interconnected network. The new vasculature is then corrected through remodeling and pruning; the nutritional demands give rise to small and large vessels, whereas local levels of oxygen and VEGF determine apoptosis of some ECs to accomplish the optimal vascular density.^[^ ^17^, ^50^ ^]^

The blood vessel formation process is governed by several biomolecular factors. The hypoxic conditions and increased lactate levels, characteristic for the hematoma, and callus at the site of an injury, stabilize expression of hypoxia‐inducible factor 1 alpha (HIF1‐*α*).^[^ ^15^, ^19^, ^20^, ^44^ ^]^ According to some studies conducted in mice, the increased expression of HIF1‐*α* was associated with hyper‐vascularization, while its depletion resulted in delayed callus formation.^[^ ^19^ ^]^ HIF1‐*α* drives expression of VEGF, which promotes both the angiogenic and vasculogenic processes,^[^ ^19^, ^20^ ^]^ and more than 60 factors related to adaptation in hypoxic conditions.^[^ ^51^ ^]^ VEGF is secreted by many cells, including MSCs, osteoblasts, hypertrophic chondrocytes, but also inflammatory cells.^[^ ^18^ ^]^ It stimulates proliferation and sprouting of ECs and its expression attracts EPCs toward the site of injury. Studies have shown that inhibition or deficiency of VEGF is reflected in the reduced angiogenic potential, healing ability, and quality of the newly formed tissue.^[^ ^15^, ^19^, ^20^, ^39^, ^44^ ^]^ Platelet EC adhesion molecule (PECAM1), also known as cluster of differentiation 31 (CD31), is an adhesion and signaling molecule expressed by vascular cells. In coordination with other molecules, it has been demonstrated to promote the migration of ECs while also ensuring maintenance of cellular integrity in terms of proper barrier function (permeability) and cell–cell junctions.^[^ ^52^ ^]^

## 4 Frequently Used In Vitro Methods for Assessment of Sprouting Angiogenesis Induced by Inorganic Agents

In vitro assays are usually the starting point of an investigation and often represent a very simple setup lacking many physiological cues (such as mechanical strain and chemotaxis) and interaction with other cell types. Although this is considered a hindrance while aiming to simulate an accurate in vivo situation, those simple in vitro assays are advantageous for examining a direct effect of an agent on an isolated cellular system and studying individual mechanisms found in complex tissues.

The in vitro angiogenic assays are designed to emulate the process of blood vessel formation and to investigate the effect of new stimuli on the behavior of ECs at the different stages of angiogenesis. The cellular response is tested for the proliferative, migrating, and sprouting capacity, attachment, morphology, viability, and phenotype commitment. The angiogenic assessment should cover multiple stages of the vessel formation process and consider the biomolecular complexity and selectivity, in which only specific factors and/or behavioral aspects are promoted/suppressed and how those events are coupled.^[^ ^53^ ^]^

The articles yielded from the literature were also screened for the different angiogenic assessment methods, which were summarized and compared in terms of their approach (direct (D) culturing of cells on the material's surface, indirect (I) culturing of cells in the material's extract) usability, reliability, costs, and general (dis)advantages (**Table** **2**).

**Table 2:** Overview and comparison of methods frequently used for assessment of angiogenic behavior of ECs

Examined property	Methods	Assays	Description of the assay	Advantages/disadvantages	Reference
Matrix degradation	Assessing MMP activity	Zymogen assay	The MMPs activity is assessed through their ability to degrade/digest gel; after staining the hydrolyzed areas appear clear in contrast to the dark background.	Inexpensive; time‐consuming	^[^ ^54^ ^]^
		Matrix invasion assay	Using a transwell with basal membrane occulated pores.	Time‐consuming	^[^ ^66^ ^]^
Proliferation	Direct cell count (staining)	DAPI	The stain labels all cells and the cell number can be evaluated from images.	Inexpensive; time‐consuming; operator errors.	^[^ ^67^ ^]^
		Trypan Blue	Labeling all cells with a fluorescent dye and counting in a haemocytometer or trypsinized and counted in a cell counter.	Possible machine errors depending on the cell density.	^[^ ^54^ ^]^
		Propidium iodide	PI labels all cells which can be then counted in a flow cytometer.	Simple; it does not indicate the ratio of live/dead cells.	^[^ ^68^ ^]^
	DNA synthesis quantification through mitotic divisions	[^3^H] thymidine	Incorporation of a labeling compound into the DNA strings and measuring the output signal (intensity) in a device with an adequate detector.	Radioactive; slow.	^[^ ^57^ ^]^
		BrdU		Not radioactive (environmentally friendly); more costly.	^[^ ^69^, ^70^ ^]^
		EdU		Click chemistry—no degradation needed for detection.	^[^ ^71^ ^]^
	Colorimetric assays	MTT	Detecting the intensity of a compound product reduced by active mitochondria.	Simple; ambiguous interpretation of data—need for an additional assay to validate the results.	^[^ ^73^, ^74^ ^]^
		Alamar Blue/PrestoBlue			^[^ ^68^, ^75^ ^]^
Migration	Migration along a gradient (chemotaxis)	Transwell assay	Monitoring cells migrating through a porous membrane toward a stimulus; standard use of 8 µm pores for HUVECs.	Easy quantification; higher costs.	^[^ ^76^, ^77^ ^]^
		Under‐agarose assay	A well with cells separated from a well with chemoattractant; the cells migrate under agarose gel toward the attractant.	Less sensitive; cheap; difficult to quantify.	^[^ ^54^ ^]^
	Wound closure capacity (chemokinesis)	Wound healing/scratch assay	Scraping a confluent layer and monitoring the closure capacity of cells.	Difficult quantification (uneven size and boundaries of the scratch).	^[^ ^78^, ^79^ ^]^
Tube formation/sprouting	Sprouting in 2D	Sprouting in Matrigel/collagen/laminin/fibrin	Plating wells with an appropriate gel and seeding cells on top; assessing length and number of sprouts/tubes/rings in a microscope.	Matrigel is relatively expensive; 2D is not an accurate representation of the in vivo situation; simple method.	^[^ ^82^, ^83^ ^]^
	Sprouting in 3D	Sprouting in a thicker basement membrane	Use of a thicker or multi‐layer setup allowing both horizontal and vertical migration; assessing length and number of sprouts/tubes/rings in a microscope.	More accurate to in vivo situation; difficult quantification.	^[^ ^72^ ^]^
Phenotype differentiation	mRNA expression	RT‐qPCR	Quantitative detection of coding sequences of angiogenesis related proteins in the DNA (VEGF, HIF1‐alpha, and PECAM1/CD31).	Time‐consuming; less sensitive.	^[^ ^85^, ^86^ ^]^
	Protein expression	ELISA, Western blot	Quantification of angiogenesis‐related proteins detected in the culture supernatant (VEGF, HIF1‐alpha) or on the membrane (PECAM1/CD31).	Sensitive methods; ELISA is simpler and cheaper than Western blot.	^[^ ^69^, ^87^ ^]^
	Other	NO release	Detection of nitric oxide, which is indicative of well‐functioning endothelium.	Quick; difficult detection due to short half‐life of NO.	^[^ ^91^, ^92^ ^]^
Indirect effect	Coculture	MSCs, osteoblasts, and chondrocytes	Culturing multiple cell types together and observing their interaction and response to different agents introduced in the culture.	Challenging culturing technique; a more accurate representation of the in vivo interactions.	^[^ ^16^, ^67^ ^]^
Adhesion/morphology	Observation of cells	Morphology and spreading	Observing cellular response to the substrate extracts/surface.	Simple and quick.	^[^ ^88^ ^]^

The next subsections provide an overview of the different types of ECs and describe the in vitro assays that study endothelial behavior and the different stages of angiogenesis.

### 4.1 Type of Endothelial Cells

Together with smooth muscle cells, ECs represent the fundamental structural units of the vascular system. They line the lumen of blood vessels and are therefore employed in the in vitro models for angiogenic assessment. There are several types of ECs that can be used in in vitro models intended to study angiogenesis. Naturally, their origin (human or animal) determines their phenotype, size (10–20 µm in diameter), and morphology; the cells differ in expression and release of biomolecular factors and in the tendency to form tissue‐specific structures, such as sprouting ability.^[^ ^54^, ^55^, ^56^, ^57^ ^]^ Therefore, the cell type should be selected according to the desired outcome of the experiment, and results should be interpreted with care.^[^ ^57^ ^]^

Primary cells are strongly preferred for assessment of angiogenesis, albeit the use of immortalized cell lines is also possible. Primary cells are isolated from a donor tissue without being subjected to any modifications and therefore represent a more accurate approximation of the actual tissue. They pose some disadvantages such as differences between individual batches and limited passage‐dependent proliferation capacity. The most commonly used human primary cells are human umbilical vein ECs (HUVECs). HUVECs are relatively large cells obtained from the endothelium of an umbilical vein. They are easy to isolate and harvest, highly proliferative, and capable of forming capillaries. For those reasons, they are frequently chosen for studying the angiogenic behavior of ECs, although they are not of microvascular origin.^[^ ^53^, ^54^, ^55^, ^56^, ^57^ ^]^ Besides HUVECs, human arterial ECs (HAECs) are another type of macrovascular ECs, suitable for models studying pathological vascular disorders.^[^ ^57^ ^]^ Among other commonly used primary ECs are human microvascular ECs,^[^ ^57^ ^]^ which are suitable for studying endothelium and neoangiogenesis in the surroundings of tumorous tissue, bovine aortic ECs,^[^ ^53^, ^54^ ^]^ and human dermal microvascular ECs.^[^ ^53^, ^54^, ^55^ ^]^

An immortalized cell line, on the other hand, is mostly established from a single cell where all cells possess identical genetic information.^[^ ^58^, ^59^ ^]^ Cell lines can be used for initial experiments assessing cytotoxicity and chemical biocompatibility of various molecules, however, due to their atypical behavior, which must be taken into consideration during experiments, they are not suitable for advanced steps of the research process.^[^ ^60^ ^]^ Unlike primary cells, they do not lose their proliferative ability after several passages. Therefore, they are not a representative sample for assessing cellular proliferation.^[^ ^58^, ^61^ ^]^ An example of an immortalized cell line is EA.hy926, a human umbilical vein cell line with a differentiation profile suitable for angiogenic in vitro models.^[^ ^62^, ^63^ ^]^

The conditions for the culture of ECs are specified by the manufacturer who usually supplies or advices appropriate culturing media. Cell line EA.hy926 can be cultured in Dulbecco's Modified Eagle Medium (Lonza) supplemented with fetal bovine serum, streptomycin, and penicillin.^[^ ^64^, ^65^ ^]^ Primary HUVECs supplied from Lonza, however, require use of specific media (endothelial cell basal medium) enriched by a number of growth factors such as VEGF, FGF, and epidermal growth factor, also offered by Lonza.

### 4.2 Assessment of Basal Membrane Degradation

Degradation of the basal membrane is a critical step, which liberates the ECs from the tightly ordered monolayer and allows for proceeding with proliferation and migration, and the eventual generation of a new capillary network. Methods for assessment of the first stage of angiogenesis, the degradation of the basal membrane, are based on measuring the activity of MMPs produced by the ECs. Those enzymes are responsible for digestion of the membrane and liberating the ECs from the blood vessel wall.^[^ ^17^, ^54^ ^]^

In a gel zymogen assay, a gelatinous substrate, such as collagen, fibrinogen, or gelatine, is embedded (co‐polymerized) in a polyacrylamide gel. Collected supernatants from the ECs, cultured for a defined period of time, are then electrophoresed through the prepared gel. For evaluation of the protease activity, Coomassie staining is utilized to detect remaining protein, with the proteolyzed regions in the gel appearing clear against the dark background.^[^ ^53^, ^54^ ^]^

Another assay enabling to assess the degradative capability of ECs is the matrix invasion assay. Cells are placed in a transwell system. The chamber membrane, which normally permits the permeation of cells through its pores, is occluded with an extract of the basal membrane; cells cannot migrate through unless they degrade the extract and thereby free the pores. The quantification of cells migrated into the lower chamber is proportional to the proteolytic activity of the cellular enzymes.^[^ ^54^, ^66^ ^]^

Both assays are relatively time‐consuming and were not found among the most frequently used assays in the reviewed papers.

### 4.3 Cell Proliferation

The reproductive capacity of stalk cells determines the growth rate of establishing capillaries. Hence, it is fundamental to analyze the effects of the biomaterial and/or its extract on the proliferative capacity of ECs. There are many assays available, which can deliver information about cell proliferation. They can be divided into categories, based on the principle they utilize to determine the cell proliferative capacity.

#### 4.3.1 Cell Staining and Counting

Cells are usually cultured for several hours/days in the presence of the investigated (potential) stimulus. Upon reaching the defined time points, a suitable staining technique can be chosen based on the desired information.

The most commonly employed fluorescent stain is 4′,6‐diamidino‐2‐phenylindole (DAPI),^[^ ^67^ ^]^ which binds to adenine‐thymine‐rich regions and is dependent on the permeability of the cellular membrane (fixed/dead cells). DAPI can be used for quantification of all cells present in individual wells, in which case it requires fixing of the cell culture with an appropriate fixative (e.g., formaldehyde). An alternative to DAPI is propidium iodide (PI),^[^ ^68^ ^]^ which also binds to the DNA of all fixed/dead cells, with little sequence preference.

Without fixing, DAPI or PI can permeate dead cells only. In combination with another fluorescent dye, that can stain live cells (e.g., Hoechst, calcein), DAPI or PI can be used for live/dead staining.

The quantification of the stained cultures can be completed by automated/manual analysis of images obtained from a microscope, or by using a flow cytometer. Another option is labeling the cells with Trypan Blue and counting them in a haemocytometer or an automated cell counter.^[^ ^54^ ^]^

#### 4.3.2 Quantification of DNA Synthesis

More advanced albeit more time consuming and costly techniques use fluorescent or radioactive labels to monitor the mitotic division of cells. A labeling agent ([^3^H] thymidine,^[^ ^57^ ^]^ bromodeoxyuridine (BrdU),^[^ ^69^, ^70^ ^]^ 5‐ethynyl‐2′‐deoxyuridine (EdU)^[^ ^71^ ^]^) is added to the culture and during subsequent cellular divisions, it becomes incorporated into the DNA. The output signal can then be monitored throughout a defined period of time in a device with an appropriate detector. The quantification of incorporated thymidine requires the use of radiation, which is proportional to the division rate and can be measured in a scintillation counter. BrdU is a newer analogue of thymidine and uses immunochemical detection methods. The most advanced EdU incorporation technique uses click chemistry instead of an antibody and unlike BrdU does not require denaturation of the DNA for detection of the signal. Both a flow cytometer and a fluorescent microscope can be used for the detection and evaluation of the proliferative capacity of cells.^[^ ^53^, ^54^, ^57^, ^71^, ^72^ ^]^

#### 4.3.3 Colorimetric Assays

Colorimetric assays employ specific compounds that can be modified by chemical reduction through mitochondria of active cells and of which their fluorescently reduced products can be measured using spectrophotometry. The output signal is often correlated with the number of active cells and is used to reflect the proliferation rate of the culture. The compound is added to the cell culture at the end of a defined cultivation period. It is usually incubated with the cells for about 1–2 h (could be more in case of slowly dividing cells), and the intensity can then be analyzed on an absorbance‐ or a fluorescent‐based plate reader. The most commonly used colorimetric assays are MTT^[^ ^73^, ^74^ ^]^ (reduction of yellow tetrazolium salt to purple formazan), Alamar Blue, and PrestoBlue^[^ ^68^, ^75^ ^]^ (reduction of blue resazurin to red resorufin).^[^ ^53^, ^54^, ^72^ ^]^

### 4.4 Cell Migration

Assays reflecting the motility and guidance of tip cells during sprouting angiogenesis can be divided into two categories: a) response and attraction of ECs toward an environmental factor along its concentration gradient (chemotaxis), b) general cellular motility (chemokinesis), and ability of cells to restore a disrupted monolayer (heal the emulated wound) after being introduced to a (potentially) proangiogenic agent.

One of the most frequently employed experimental setups of the first group is the transwell:^[^ ^76^, ^77^ ^]^ a two‐chamber system with a separative cell‐permeable membrane. Precultured cells are placed in the upper chamber (the well insert), while the medium containing the active agent is in the lower chamber. The cells are then incubated for several hours. The size of the pores in the membrane requires active adjustment of cell morphology to allow the cells to pass through. Afterward, the cells are fixed, stained, and the number of cells migrated through the membrane is analyzed.^[^ ^54^, ^55^, ^57^, ^72^ ^]^ Another assay intended for the evaluation of cellular chemotaxis is the under‐agarose assay. In this experimental setup, the cells migrate from one well toward a well with an attractant under the agarose gel, which separates the wells.^[^ ^54^ ^]^

The general cellular motility can be assessed through a wound‐healing/scratch assay.^[^ ^78^, ^79^ ^]^ First, cells are cultured until confluence is reached after which the monolayer is scratched. The cells are usually monitored for several hours and pictures are taken at defined time points. The wound healing capacity can then be evaluated from the pictures using an ImageJ plugin (MRI wound healing tool).

Cell starvation in a serum‐free medium prior to those experiments is a common practice to maximize the migratory and motility response of ECs.^[^ ^54^, ^80^, ^81^ ^]^

### 4.5 Tube Formation/Sprouting

The ability of ECs to organize into tube‐like structures is the first visual indication of establishing capillary lumen and the new vascular network. In principle, it can be tested in several gel‐containing models.^[^ ^82^, ^83^ ^]^

Generally, wells intended for cell seeding are coated with one of several available substrates (collagen, laminin, fibrin, and Matrigel) and allowed to solidify. Subsequently, cells are seeded onto the gel and their sprouting behavior is monitored for several hours. Microscope imaging can then be used for evaluation of the ECs sprouting ability. The most common methods for quantification are counting the number of nodes/branches/sprouts or the length of rings/tubes formed. Such an analysis can be done either manually or fully automated.^[^ ^84^ ^]^

Unlike other substrates, Matrigel contains many growth factors, enhances attachment, and highly stimulates migration and differentiation of cells. The potential overstimulation has been demonstrated by seeding other non‐vascular cell types (e.g., fibroblasts) into Matrigel, which were also able to form tubular structures, although it does not belong to their normal behavioral features. Hence, the use of growth factor‐reduced Matrigel is strongly recommended for the assessment of sprouting.

To approximate the 3D in vivo conditions and allow for both horizontal and vertical migration and sprouting of cells, the thickness of the coated layer can be increased, cells can be mixed with the gel or seeded between gel layers.^[^ ^53^, ^54^, ^57^, ^72^ ^]^

### 4.6 Gene/Protein Expression

The type and concentration of specific biomolecules expressed by ECs reflect their angiogenic behavior. In addition, the maturity and phenotype specificity of ECs can be determined through the detection of specific types of gene and protein expression. When assessing the angiogenic capacity of ECs, the most frequently tested angiogenic factors are VEGF, HIF1‐*α*, and PECAM1 (CD31).

To determine the types and quantities of activated genes, reverse transcription‐quantitative polymerase chain reaction (RT‐qPCR) is performed.^[^ ^85^, ^86^ ^]^ In this method, extracted and reversely transcribed genetic information is multiplied, bound to a detectable molecule (a fluorescent marker), and analyzed in a qPCR machine.

Enzyme‐linked immunosorbent assay (ELISA) is a commonly used diagnostic tool capable of detecting synthesized or secreted proteins in vitro.^[^ ^87^ ^]^ The method utilizes protein‐specific antibodies and fluorescent substrates (added in defined order) that enable quantification of the existing proteins using a spectrophotometer. Protein concentration and/or its subcellular localization can be quantified using Western Blotting,^[^ ^69^ ^]^ where proteins are denatured prior to their loading into an electrophoretic gel setup.^[^ ^55^, ^56^ ^]^

### 4.7 Other Assays

#### 4.7.1 Attachment, Morphology, and Viability

Although evaluation of the cellular attachment is not directly assigned to any of the stages of sprouting angiogenesis, it is often included in the angiogenic assessments, as proper attachment and morphology are considered to be pivotal for further angiogenic development stages.^[^ ^88^ ^]^ Monitoring of cells cultured with (extracts of) a given biomaterial can provide information about their response to its (bio)chemical composition. To observe and evaluate the behavior of cells influenced by both chemical and physical properties, cells can be seeded directly onto the biomaterial surface. The most common methods to assess the morphology, spreading, and viability of ECs include scanning electron microscopy (SEM) and confocal laser scanning microscopy.^[^ ^83^, ^87^, ^89^ ^]^

#### 4.7.2 NO Production

Nitric oxide (NO) is the most important substance produced by the endothelium. Proper functioning of ECs is dependent on balanced levels of NO, and their disruption is associated with severe impairments of the vascular system (vasoconstriction, inflammation, and atherosclerosis).^[^ ^51^, ^90^ ^]^

Measuring the produced NO is a good way to gain information about the quality of the established endothelium.^[^ ^91^, ^92^ ^]^ Its very short half‐life led to the development of methods based on colorimetric or fluorometric detection. They utilize the rapid oxidation of NO and subsequent enzymatic conversion of the nitrate to nitrite by nitrate reductase to form a colored quantifiable product (Griess test), which can be analyzed on a plate reader or other suitable detector.^[^ ^93^ ^]^

#### 4.7.3 Coculture with Other Cell Types

Depending on the intended application, ECs are often cultured in the presence of other cells to emulate the in vivo conditions and the mutual biological interactions.

For bone tissue engineering purposes, ECs are most frequently cultured with MSCs or osteoblasts, which secrete VEGF and other specific proteins to facilitate the differentiation of ECs (increased expression of CD31) toward angiogenesis.^[^ ^94^, ^95^, ^96^ ^]^

Various approaches taken for the evaluation of the interactions were identified. The response of ECs to the growth factors secreted by other cell types, such as MSCs, can be studied in a simple culture setup combining the endothelial culture medium with the conditioned medium from the other cell type.^[^ ^16^ ^]^ Introducing a second cell type significantly increases the complexity of the experimental setup.^[^ ^67^ ^]^ The additional challenges encountered in these models are mostly related to the seeding protocol, establishment of a proper media composition required for the survival of included cells and the ratio of seeded cells.

Many publications have established protocols with recommended cell number ratios and temporal order in which ECs and MSCs/osteoblasts should be seeded. The readers are advised to seek detailed guidance for those assays elsewhere (e.g.,^[^ ^96^, ^97^, ^98^ ^]^) as it is beyond the scope of this review.

## 5 Interaction of Endothelial Cells with Inorganic Angiogenic Agents

### 5.1 Copper

Copper is known for its antibacterial activity and angiogenic potential.^[^ ^13^, ^87^ ^]^ An optimal concentration of copper has been also shown to stimulate normal bone metabolism and reduce the bone resorption rate.^[^ ^99^, ^100^ ^]^ Therefore, the element represents nowadays an attractive choice for general tissue engineering solutions, including bone regeneration.

Seventeen articles were identified in the literature and included in the comparison (**Table** **3**). The largest group of biomaterials that incorporated copper were bioceramics.^[^ ^67^, ^74^, ^82^, ^91^, ^101^, ^102^, ^103^ ^]^ Titanium was found to be the most common metallic material used in combination with copper, due to its superior mechanical properties and excellent suitability for orthopedic applications.^[^ ^64^, ^73^, ^79^, ^87^, ^104^ ^]^ Mg‐Cu alloy represented a group of biodegradable metals and a solution for long‐lasting antibacterial effects.^[^ ^83^ ^]^

**Table 3:** Literature overview of the effects of copper on ECs

Copper							
Tested material	Effective conc./ion release Cu^2+^	In vitro cell line/in vivo species	Assays—Direct (D)/indirect (I)	Incubation time	Other material properties	Results	Ref
Cu‐Ti‐O‐titanium	SC: 4.62 at%; no IRP	EA.hy926	Cell adhesion (D); live/dead viability (D); MTT proliferation (D); NO release (D); ELISA (D); tube formation in ECMatrix (I)	0.5, 1, 4, 24 h; 1, 3, 5 d; 1, 3, 5 d; 24 h; 1 d; 4, 8, 18 h	The nanotube structure became less organized with increasing Cu content and tubular length decreased.	The Cu‐doped nanotubes increased proliferation, VEGF secretion, and tube formation.	^[^ ^64^ ^]^
Sr/Cu‐bioactive glass	SC: 0.14 at%; IRP: 0.0025 mm mg^−1^	HUVECs	MTT viability (I); tube formation in Matrigel (I)	24, 48 h; 16 h	The fiber diameter increased with Sr content.	The Cu‐dopant promoted angiogenic behavior of HUVECs.	^[^ ^74^ ^]^
Cu‐bioglass	MC: 1 wt%; IRP: ≈0.95–1.15 mg L^−1^	HDMECs	Staining for ECs surface markers (I); tube formation in Matrigel (I)	7, 14 d; 24 h	‐	The Cu‐enriched scaffold stimulated ECs toward angiogenesis through increased VEGF expression by MSCs.	^[^ ^82^ ^]^
Mg‐Cu alloy	MC: 0.03 wt%; IRP: 0.15 mg L^−1^ (after 5 d)	HUVECs; SD rats	MTT proliferation (I); cell morphology (I); scratch migration (I); tube formation in Matrigel (I); RT‐qPCR (I); Western blot (I); aortic ring model	1, 3, 5 d; 12 h; 6, 12 h; 4, 8, 16 h; 3 d; 3 d; 7, 14 d	‐	The Mg‐Cu alloy (especially with 0.03 wt%) showed stimulation toward angiogenesis, possibly owing to both Mg and Cu.	^[^ ^83^ ^]^
Cu^2+^	‐	Endothelial cells	‐	‐	‐	Enhanced proliferation of ECs by the Cu ions.	^[^ ^100^ ^]^
Cu‐bioactive glass	MC: 1.6 mol%; IRP: ≈0.7 mg L^−1^	HUVECs; chicken embryos	Tube formation in Matrigel (I); chicken chorioallantoic membrane assay	36 h; 5 d	‐	The extracts with Cu improved tubule formation in vivo and vessel formation in the ex vivo model.	^[^ ^101^ ^]^
Cu‐calcium phosphate	MC: 0.1 mol% (Cu/(Cu + Ca)); IRP: 0.098 mg L^−1^ (after 7 d)	HUVECs	CCK‐8 proliferation (D); cell attachment and morphology (D); live/dead viability (D); NO release (D); RT‐qPCR (D)	2, 4 d; 24 h; 24 h; 2 d; 7 d	Crystal size increased with Cu concentration.	The samples with 0.05 and 0.1 mol% improved the angiogenic capacity of HUVECs.	^[^ ^91^ ^]^
Cu/Si‐TiO_2_ coating	SC: 0.76 at%; IRP: 0.01 mg L^−1^ (after 7 d)	EA.hy926	Live/dead viability (D); MTT proliferation (D); cell morphology (D); ELISA (D); tube formation in ECMatrix (I)	1, 3, 5 d; 1, 4, 7 d; 1 d; 24 h; 4, 8, 18 h	‐	The M‐CuSi5 alloy with 0.76 at% Cu presented the best pro‐angiogenic properties.	^[^ ^73^ ^]^
Cu/Zn‐calcium phosphate	MC: 0.02 mol/l; IRP: 0.9 mg L^−1^ (after 7 d)	Vascular ECs (in cc w. BMSCs)	Cell morphology (D); DAPI staining (D); CCK‐8 proliferation (D); ELISA (D)	5 d; 5 d; 1, 4, 7 d; 14 d	Addition of dopant resulted in cubical nano‐/microparticles on the surface, depending on the concentration.	Cu/Zn co‐dopant system improved angiogenic capacity of HUVECs in cc with BMSCs.	^[^ ^67^ ^]^
Cu^2+^	0.06–14.1 mg L^−1^	HUVECs	Alamar Blue viability (D); scratch migration (D); intracellular ROS levels (D)	3 d; 6 h; 24 h	‐	Improved proliferation of ECs Cu of up to 222 µm improved proliferation and up to 1 µm also migration of ECs.	^[^ ^78^ ^]^
Cu‐Ti6Al4V	MC: 6 wt%; IRP: 0.75 µg cm^−2^ (after 7 d)	EA.hy926	Cell attachment and morphology (D); CCK‐8 proliferation (D); RT‐qPCR (D); ELISA (D)	1, 3 d; 1, 3, 5, 7 d; 3, 7 d; 3, 7 d	The presence of Cu resulted in micropores.	The Ti6Al4V‐6Cu alloy enhanced angiogenic properties of ECs.	^[^ ^87^ ^]^
Cu‐eluting graphene	MC: 0.36 g; IRP: 7% (missing units; after 3 d)	SVEC4–10	Proliferation (DNA quantification) (D); cell morphology (D); tube formation in Matrigel (I); RT‐qPCR (D)	3, 7 d; 24 h; 4 h; 3 d	The samples present different roughness (*R*_a_ = 0.75–2.18 µm)	The sustained Cu release from PCL/RGO Cu enhances proliferation, migration, and tube formation of ECs.	^[^ ^89^ ^]^
Cu/Ca‐bioglass‐alginate	IRP: ≈5 mg L^−1^ (after 7 d)	HUVECs; HDMECs	MTT viability (I); tube formation in Matrigel (I)	24 h; 24 h (1–2 w preculture)	‐	The presence of bioglass nanoparticles (combined with Cu^2+^) enhances the angiogenic capacity of HUVECs.	^[^ ^102^ ^]^
Cu‐HA	MC: 3.15 wt%; No IRP	Human ECs; New Zealand white rabbits	Cell adhesion and spreading (D); Alamar Blue viability (D); subcutaneous implantation	5 d; 1, 3, 5 d; 1, 4, 8 w	Addition of Cu through hydrothermal treatment resulted in micro/nanostructured surface.	The surface architecture of Cu5‐HA supported the spreading and proliferation of ECs in vitro and vessel formation in vivo.	^[^ ^103^ ^]^
N/Cu‐titanium	SC: 23 at%; IRP: 0.1 mg L^−1^ (after 7 d)	HUVECs	Alamar Blue proliferation (D); scratch migration (D)	1, 4, 7 d; 6 h (3 d preculture)	The surfaces of implanted samples were evenly smooth.	The greater number of Cu^2+^ ions released from N/Cu‐Ti promotes angiogenic behavior of HUVECs.	^[^ ^79^ ^]^
CuSO_4_	19.9 mg L^−1^	HUVECs; CD1 mice	Tube formation in Fibrin gel; subcutaneous scaffold implantation	12 d; 30 d	‐	50 µg ml^−1^ of CuSO_4_ improved the tube formation of ECs in vitro and in combination with GFs might be a good option for in vivo solutions.	^[^ ^105^ ^]^
Ca‐P‐Zn‐Cu coating on Ti	6.3 mg L^−1^ (supplemented media)	HUVECs	Tube formation in collagen gels; transwell migration	24, 48 h; 4 h		Improved migration activity with Cu (6.3 mg L^−1^) in combination with Zn, while cytotoxic effects were observed with higher Cu concentration (31.5 mg L^−1^).	^[^ ^104^ ^]^

Generally, the addition of copper to different materials resulted in increased proliferative, migration and tube formation capability, secretion of angiogenesis dependent factors (VEGF) by ECs in vitro, and favorable vessel formation, also in in vivo models. Some studies reported morphological alteration of the materials surface with the additions of different concentrations of the agent.^[^ ^64^, ^67^, ^74^, ^79^, ^87^, ^89^, ^91^, ^103^ ^]^ Along with the chemical stimulatory agents, surface morphology in the form of nanostructures, or wettability can also favor angiogenic capacity of ECs and their adhesion and spreading on the material.^[^ ^103^ ^]^

The investigation of the effect of medium‐supplemented Cu on the angiogenic behavior of HUVECs yielded data of the cellular response to various doses of the pure ion.^[^ ^78^, ^104^ ^]^ The stimulatory effects on proliferation were observed with a concentration of up to 14.1 mg L^−1^, while migration was enhanced only up to 0.06 mg L^−1^ of copper in the medium.^[^ ^78^ ^]^ Cu‐Zn supplemented medium with Cu concentration of 6.3 mg L^−1^ showed improved migration activity, while increased amount of Cu (31.5 mg L^−1^) was associated with cytotoxic effects on ECs.^[^ ^104^ ^]^ Similarly, the effect of CuSO_4_ on ECs was investigated.^[^ ^105^ ^]^ Those findings showed improvement in endothelial activity with 19.9 mg L^−1^ of Cu^2+^ (equivalent to 50 mg L^−1^ of CuSO_4_), which is somewhat higher than the above‐mentioned findings.^[^ ^78^ ^]^ The optimal concentration of Cu ions released from the materials indicated in the publications also differed. The effective range of Cu^2+^ released from the majority of bioceramics was between 0.7 and 1.2 mg L^−1^.^[^ ^67^, ^82^, ^101^ ^]^ However, lower concentrations of ions leading to a positive endothelial response were also reported, such as 0.098 mg L^−1^ of Cu^2+^ released (on day 7) from a Cu‐modified calcium phosphate cement.^[^ ^91^ ^]^ Similar release profiles favoring the ECs were detected in the case of N/Cu doped titanium where the concentration of Cu^2+^ was 0.10 mg L^−1^.^[^ ^79^ ^]^ Much higher doses of Cu^2+^ have been reported from Zn/Cu‐doped calcium phosphate^[^ ^67^ ^]^ and Cu‐crosslinked alginate with bioactive glass nanoparticles,^[^ ^102^ ^]^ with concentrations of 1.0 and 5.0 mg L^−1^ (day 7), respectively.

### 5.2 Magnesium

Magnesium is a very light and biocompatible metal. Its essential role in bone metabolism and degradability make it a promising solution for some areas of regenerative medicine demanding a new type of degradable metallic medical devices.^[^ ^14^ ^]^

Nine articles discussing the effect of Mg on ECs were identified in the search and the summary of the findings can be found in **Table** **4**. Unlike copper or strontium, magnesium was much more often incorporated within metallic materials^[^ ^69^, ^75^, ^83^, ^106^, ^107^ ^]^ rather than in bioglasses or bioceramics.^[^ ^108^ ^]^ The response of ECs to the magnesium‐containing materials varied and greatly depended on the concentration. Several studies showed improvement in angiogenic capacity in terms of proliferation, migration, tube formation, and expression of angiogenic genes, after introducing the culture to magnesium.^[^ ^69^, ^107^, ^108^, ^109^ ^]^ The effective concentrations reported were usually much higher compared to the other elements being mostly in the range of 60–122 mg L^−1^,^[^ ^75^, ^76^, ^83^ ^]^ although endothelial activation was also observed at a much lower concentration of 0.015 mg L^−1^ Mg^2+^ released from Zn/Mg‐coated titanium.^[^ ^107^ ^]^ Concentration‐dependent cytotoxicity was studied using a tricalcium phosphate (TCP) material.^[^ ^108^ ^]^ The TCP doped with 1.0 wt% Mg stimulated ECs, while a TCP scaffold with 4.0 wt% Mg had an inhibitory effect on their proliferative activity and growth. Similarly, the cytotoxic effects of untreated Mg‐Ca alloy due to excessive generation of corrosion products were discussed in another study.^[^ ^75^ ^]^ To mitigate the adverse inhibitory activity, the alloy was subjected to plasma electrolytic oxidation (PEO) treatment. Similarly, alkali heat treatment was adopted to achieve a more moderate release profile of the Mg‐Ca alloy.^[^ ^106^ ^]^

**Table 4:** Literature overview of the effects of magnesium on ECs

Magnesium							
Tested material	Effective conc./ion release Mg^2+^	In vitro cell line/in vivo species	Assays—Direct (D)/indirect (I)	Incubation time	Other material properties	Results	Ref
(Si‐)Mg‐Ca alloy	SC: 37–64 at%; IRP: 70 mg L^−1^ (after 5 d)	C166‐GFP endothelial cell line	Cell morphology (D); Alamar Blue cytocompatibility (I)	30 min; 5 d	The samples differed in surface roughness (0.7–4.3 µm), thickness, and porosity.	The untreated surface of Mg‐Ca alloy disabled the growth and proliferation of ECs.	^[^ ^75^ ^]^
Mg‐TCP scaffolds	MC: 0.6 wt%; IRP: 56 mg L^−1^ (after 1 d)	HUVECs	CCK‐8 proliferation (I); live/dead viability (I); cell morphology visualization (I); NO release (I); RT‐qPCR (I)	1, 4, 7 d; 24 h; 24 h; 48 h; 7, 14 d	‐	The scaffold with 0.6 wt% of Mg promoted angiogenic behavior of HUVECs, while 2.4 wt% inhibited them.	^[^ ^108^ ^]^
Mg‐alloy w. NO nanofibres	MC: 94 wt%; no IRP	HUVECs	WST‐1 proliferation (I); tube formation in Matrigel (I)	1, 2, 3 d; 12 h	‐	The rapid degradation of Mg did not match with the healing progress; here NO is incorporated to improve the healing process.	^[^ ^136^ ^]^
Mg‐Zn‐Mn alloy	MC: 97 wt%; No IRP	HUVECs	DNA synthesis capacity (BrdU) (I); MTT viability (I); tube formation in Matrigel (I); Western blot (I); RT‐qPCR (I)	24, 48 h; 24, 48, 72, 96, 120 h; 16 h; N/A; N/A	‐	The 6.25% Mg‐Zn‐Mn alloy extract could improve the angiogenic behavior of HUVECs, most likely owing to Mg.	^[^ ^69^ ^]^
Mg^2+^	61–122 mg L^−1^	ECs (not specified); nude mice; SD rats	Transwell migration (I); subcutaneous implantation; cranial defects	24 h; 1, 3, 7, 14 d; 4 w	‐	Mg improved angiogenic behavior of HUVECs through VEGF secretion of MSCs, and vascularization in in vivo models.	^[^ ^76^ ^]^
Mg‐acrylic bone cement	MC: 5.3 wt%; IRP: 50 mg L^−1^ (after 1 d)	HUVECs; SD rats	Tube formation in Matrigel (I); femoral defects	18 h; 2 month	‐	The Mg‐induced degradation improved tube formation of HUVECs.	^[^ ^109^ ^]^
Mg‐Ca alloy	SC: 10–12 at%; No IRP	ECV304	Cell adhesion and morphology (D); CCK‐8 proliferation (D)	6, 24 h; 24 h	‐	The modification improved the corrosion rate and cytocompatibility of the Mg alloy.	^[^ ^106^ ^]^
Zn/Mg‐titanium	IRP: 0.015 mg L^−1^ (after 7 d)	HUVECs	CCK‐8 proliferation (D); RT‐qPCR (D); immunofluorescence analysis (D); intracellular Zn detection	1, 4, 7 d; 10 d; 10 d; 10 d	‐	The presence of Mg showed proangiogenic effects (proliferation, gene expression).	^[^ ^107^ ^]^
Mg‐Cu alloy	MC: 99 wt%; IRP: ≈190 mg L^−1^ (after 5 d)	HUVECs; SD rats	MTT proliferation (I); cell morphology (I); scratch migration (I); tube formation in Matrigel (I); RT‐qPCR (I); Western blot (I); aortic ring model	1, 3, 5 d; 12 h; 6, 12 h; 4, 8, 16 h; 3 d; 3 d; 7, 14 d	‐	The Mg‐Cu alloy showed stimulation toward angiogenesis, possibly owing to both Mg and Cu.	^[^ ^83^ ^]^

### 5.3 Silicon

Silicon is a major component of bioglasses and bioceramics. For its capacity to stimulate both MSCs/osteoblasts toward osteogenesis and ECs toward angiogenesis, silicon is utilized for many tissue engineering applications.^[^ ^14^, ^110^ ^]^

Within this review, 16 articles were identified and included in the comparison presented in **Table** **5**. The findings show that silicon was often used in combination with a titanium alloy and incorporated within its surface.^[^ ^65^, ^73^, ^92^, ^111^, ^112^, ^113^, ^114^, ^115^ ^]^ Such a solution exhibits good mechanical properties imparted by titanium and utilizes bioactive osteogenic/angiogenic component in the form of silicon ions/particles.^[^ ^7^ ^]^ The addition of silicon often resulted in an alteration of the surface morphology. Decreasing roughness with the addition of silicon was reported^[^ ^92^, ^112^ ^]^ as well as improved wettability.^[^ ^65^, ^113^ ^]^ Bioceramics often incorporate silicon directly in their matrices. A frequently reported bioceramic was Ca–Mg–Si,^[^ ^116^, ^117^ ^]^ a combination of three known bioactive components, or silicon‐containing hydroxyapatite.^[^ ^118^ ^]^

**Table 5:** Literature overview of the effects of silicon on ECs

Silicon							
Tested material	Effective conc./ion release Si^4+^	In vitro cell line/In vivo species	Assays—Direct (D)/indirect (I)	Incubation time	Other material properties	Results	Ref
Ti‐Si‐N coating on Ti6Al4V	SC: 20 at%; No IRP	EA.hy926	CCK proliferation (D); cell morphology and spreading (D); NO release (D)	1, 5 d; 5 d; 5 d	Decreasing nanoroughness with increasing Si content may affect the attachment properties.	Better morphology and greater spreading, increased proliferation and endothelialisation.	^[^ ^92^ ^]^
Si‐micro/nano‐structured titanium	SC: 0.86 at%; IRP: 23 mg L^−1^; (after 7 d)	EA.hy926	Cell adhesion (D); actin assay (D); cell morphology (D); live/dead viability (D); MTT proliferation (D); ELISA (D); tube formation in EC Matrix (I); RT‐qPCR (D)	0.5, 1, 4 h; 1, 4, 24 h; 1 d; 1, 3, 5 d; 1, 4, 7 d 24 h; 4, 8, 15 h; 3 d	Micro‐ and nanostructures from MAO and HT treatment respectively influenced the cell adhesion and the Si release profile.	Nanostructures secured a more constant Si release profile and improved the angiogenic behavior of HUVECs.	^[^ ^111^ ^]^
Ti‐Si‐N coating on Ti6Al4V	SC: 12 at%; No IRP	EA.hy926	NO release (D); cell morphology and spreading (D)	3 d; 24 h	Decreasing nanoroughness with increasing Si content.	Enhanced adhesion of endothelial cells on the coating.	^[^ ^112^ ^]^
Silk fiber w. Zn + Si‐BrC brushite	MC: 0.5 wt%; No IRP	Porcine ECs; New Zealand white rabbits	Tube formation in collagen (D); Alamar Blue proliferation (D); viability assay with PI (D); NO release (D); femur defect	N/A; 1, 3, 7 d; 7 d; 1, 7 d; 1, 3 month	‐	Positive effect of Si (and synergistic effect of Si/Zn) on angiogenesis.	^[^ ^68^ ^]^
Bioactive glass nanoporous structure	MC: 40 mol% (85 mol% SiO_2_); IRP: 21 mg L^−1^ (after 7 d)	HUVECs; SD rats	Scratch migration (I); tube formation in Matrigel (I); subcutaneous implantation	24 h; 3, 6 h; 2, 4 w	Nanofibrous structure enhances neo‐blood vessel formation.	Stable delivery of Ca and Si and their synergistic effect with the nano‐sites of improved angiogenesis.	^[^ ^120^ ^]^
Si‐DLC coating on Ti6Al7Nb	SC: 14–22 at%; No IRP	EA.hy926	Live/dead viability (D); XTT viability (I,D)	48 h; 48 h	Increasing wettability with higher Si content.	Si is tolerated by cells up to the limit between 14 and 22 at%.	^[^ ^113^ ^]^
Si‐TiO_2_ nanotubes	SC: 2.8 at%; IRP: 7 mg L^−1^ (after 1 d)	EA.hy926	Live/dead viability (D); tube formation in ECMatrix (I); NO release (I); ELISA (I)	1, 3, 5 d; 4, 7, 17 h; 24 h; 24 h	Increase of Si content increases the hydrophilicity.	The incorporation of Si into the material boosted the angiogenic capacity of ECs.	^[^ ^65^ ^]^
Strontium‐HT‐Gahnite	1.6–6.6 mg L^−1^ (diluted extracts)	HUVECs	MTT proliferation (I); transwell migration (I); RT‐qPCR (I); calvarial defect	1, 4, 7 d; 18 h (7 d preculture); 4 d; 4–6 w	‐	Increased metabolic activity at day 7, migration capacity, and mRNA expression of HUVECs with the dissolution products.	^[^ ^77^ ^]^
Ti‐Si‐N coating on titanium	SC: ≈11–13 at%; No IRP	EA.hy926	Cell morphology and spreading (D); CCK‐8 proliferation (D); NO release (D); RT‐qPCR (D); Western blotting (D)	24 h; 1, 6 d; 6 d; 6 d; N/A	‐	Si promoted endothelial proliferation and upregulates VEGF in ECs.	^[^ ^114^ ^]^
Si‐TiO_2_	SC: 1.8 wt%; IRP: 3.5 mg L^−1^ (after 7 d)	HUVECs	Alamar Blue proliferation (D); cell morphology, live/dead viability (D); scratch migration (D); tube formation in Matrigel (I); ELISA (D); RT‐qPCR (D)	1, 4, 7 d; 7 d; 8 h (1 d preculture); 12 h; 1, 3, 5, 7 d; 4, 7, 14 d	‐	The coating with 1.8 wt% of Si improved the proliferation, migration, and VEGF, tube formation of HUVECs.	^[^ ^115^ ^]^
Mesoporous silica microspheres	IRP: ≈22 mg L^−1^ (after 7 d)	HUVECs; domestic chicken embryos	CCK‐8 proliferation (I); RT‐qPCR (I); Western blotting (I); immunohistochemistry (I); tube formation in Matrigel (I); scratch migration (I); transwell migration (I); angiogenesis in chick chorioallantoic membrane (CAM)	1, 3, 7 d; 24 h; 24 h; 24 h; 0, 4, 6, 12 h; 12, 24 h; 12 h; 11 d	‐	The presence of Si promoted angiogenic capacity of HUVECs through stimulating expression of HIF1‐alpha, especially in combination with the delivery of VEGF.	^[^ ^119^ ^]^
Si‐oxynitro‐phosphide coating	SC: 53–62 at%; No IRP	HUVECs	Cell attachment (D); MTS viability (D); MTS growth (D); proliferation with Calcein‐AM (I); transwell migration (I); matrix deposition (D); tube formation in Matrigel (D); RT‐qPCR (D)	4 h; 24 h; 1, 3, 7 d; 24, 48 h; 24 h; 5 d; 6 h; 24, 72 h	Surface wettability correlated with the number of attached cells.	The silica‐based coatings enhanced proliferation, migration, matrix deposition, and tube formation VEGF expression of HUVECs.	^[^ ^137^ ^]^
Cu/Si‐TiO_2_	SC: 16 at%; IRP: ≈27 mg L^−1^ (after 7 d)	EA.hy926	Live/dead viability (D); MTT proliferation (D); cell morphology (D); ELISA (D); tube formation in ECMatrix (I); RT‐qPCR (I)	1, 3, 5 d; 1, 4, 7 d; 1 d; 24 h; 4, 8, 18 h; 3 d	‐	The implant with 16 at% of Si showed the best proangiogenic property by stimulating the proliferation, favorable morphology, and gene expression of ECs.	^[^ ^73^ ^]^
Ca–Mg–Si bioceramics	1.18–4.44 mg L^−1^ (diluted extracts)	HAECs	WST‐1 proliferation assay (I); NO release (I); tube formation in ECMatrix (I); RT‐qPCR (I)	4 d; 24 h; 2.5, 5.5, 17 h; 4 d	‐	Ceramics releasing higher amount of Si had greater stimulatory effect on angiogenic behavior of ECs.	^[^ ^116^ ^]^
Ca–Mg–Si bioceramics	0.6–2.1 mg L^−1^ (diluted extracts)	HAECs; New Zealand rabbits	WST‐1 proliferation (I); tube formation in ECMatrix (I); RT‐qPCR (I) NO release (I); scaffold implantation near distal femur	4 d; 2.5, 5.5, 17 h; 4 d 24 h; 8, 16 w	‐	Presence of Si stimulated angiogenic behavior of ECs in vitro and neovascularization in vivo.	^[^ ^117^ ^]^
Si‐HA	SC: 6.15 at%; IRP: 17 mg L^−1^ (after 7 d)	HUVECs; white leghorn chicken eggs; Wistar rats	Viability with Calcein AM (D); cell adhesion (D); proliferation with PicoGreen (D); NO release (D); ELISA (D); chicken chorioallantoic membrane assay; subcutaneous implantation	24 h; 24 h; 1, 7 d; 1, 7 d; 1, 7 d; 4 d; 2 w	‐	Scaffold with Si had stimulatory effects on functionality and viability of ECs.	^[^ ^118^ ^]^
(Si‐)Mg‐Ca alloy	SC: 10 at%; IRP: 2.0 mg L^−1^ (after 5 d)	C166‐GFP EC line	Cell morphology (D); Alamar Blue cytocompatibility (I)	30 min; 5 d	The samples differed in surface roughness (0.7–4.3 µm), thickness, and porosity.	The Si topography promoted the cellular organization.	^[^ ^75^ ^]^

The literature findings showed that silicon is capable to effectively promote the angiogenic behavior of ECs by increasing their proliferation, migration capacity, enhancing the tube formation process, and upregulating the expression of angiogenesis‐related genes (VEGF, HIF1‐*α*). The optimal concentration varied among the studies but could generally be divided into a low concentration and a high concentration group. Favorable concentrations between 1.0 and 7.0 mg L^−1^ were reported,^[^ ^65^, ^75^, ^77^, ^115^, ^116^, ^117^ ^]^ while enhanced ECs activity in a concentration range of 17–27 mg L^−1^ was also observed.^[^ ^73^, ^111^, ^118^, ^119^, ^120^ ^]^

### 5.4 Strontium

Strontium and its role in the bone formation process have been addressed by many studies. The robust capacity of this element to stimulate osteoblast differentiation and promote formation of new bone tissue represents a promising solution for orthopedic implants, granting a stronger attachment between to the implant.^[^ ^16^, ^28^, ^121^, ^122^, ^123^ ^]^ However, despite the importance of angiogenesis in the fracture healing process, the effect of strontium on ECs has not been widely investigated.

This review yielded 14 articles discussing the effect of strontium on ECs (**Table** **6**). The employed materials ranged from ceramics and metals to polymer matrices. Strontium was incorporated either in the bulk material or on its surface (either as an ion or nanoparticle), which determines its release profile characteristics.

**Table 6:** Literature overview of the effects of strontium on ECs

Strontium							
Tested material	Effective conc./ion release Sr^2+^	In vitro cell line/in vivo species	Assays—Direct (D)/indirect (I)	Incubation time	Other material properties	Results	Ref
SCPP	MC: 8 mol%; No IRP	HUVECs (cc w. OB); New Zealand white rabbits	MTT proliferation (D); tube formation (I); ELISA (D); in vivo implantation (D)	7, 14, 21, 28, 35 d; N/A; 28 d; 4, 8, 16 w	SCPP (presence of Sr) demonstrated much smoother surface than CPP and HA.	Better angiogenic properties of SCPP than CPP and HA.	^[^ ^126^ ^]^
Sr‐TiO_2_ nanoporous surface	IRP: 0.6 mg L^−1^ (after 7 d)	HUVECs (CM from BMSCs); Beagle dogs	Transwell migration (I); tube formation in ECMatrix(I); in vivo implantation (D)	24 h; 24 h; 6 w	‐	TiO_2_ coating promoted angiogenic potential of BMSCs and HUVECs (conditioned medium from BMSCs).	^[^ ^16^ ^]^
Sr‐TiO_3_ nanotubes	SC: 12.5 at%; IRP: 1.4–1.5 mg L^−1^ (after 1 d)	EA.hy926 (CM from OB)	NO release (I); tube formation ECMatrix (I)	24 h; 4, 8, 18 h		More NO production and tube formation with strontium.	^[^ ^131^ ^]^
Sr‐graphene ox.‐collagen scaffold	IRP: 45% (no units)	HUVECs (CM from hADSC); Rats	Viability, morphology, adhesion (D); transwell migration (I); tube formation in Matrigel (I); cranial defect	24 h; 24 h; 6 h; 4, 12 w	Sr‐GO‐Col exhibited rougher surface than collagen.	Vascularization potential improved by Sr‐GO‐Col.	^[^ ^88^ ^]^
Strontium ranelate	7.47 mg L^−1^ (medium with Sr)	HUVECs	Transwell migration (D); tube formation in Matrigel (D); Western blotting (D); RT‐qPCR (D)	24 h; 4–12 h (48 h preculture); 0, 15, 30, 60, 90 min	‐	Better migration and more branching points and loops detected with SrR.	^[^ ^124^ ^]^
Strontium‐HT‐Gahnite	0.24–0.96 mg L^−1^ (diluted extracts)	HUVECs; SD rats	MTT proliferation (I); transwell migration (I); RT‐qPCR (I); calvarial defect	1, 4, 7 d; 18 h (7 days preculture); 4 d; 4–6 w	Angiogenic Si incorporated in the material.	Increased metabolic activity at day 7, migration capacity and mRNA expression of HUVECs with the dissolution products.	^[^ ^77^ ^]^
Sr‐calcium silicate	≈1.1–4.2 mg L^−1^ (diluted extracts)	HUVECs; Fisher 344 rats	MTT proliferation (I); tube formation in ECMatrix (I); calvarial defects	1, 3, 7 d; 4, 8, 12 h; 4 w	‐	Greater proliferation after 7 days and higher stimulation toward tube formation with SrCS. Better vascularization of newly formed bone.	^[^ ^130^ ^]^
Sr‐TiO_2_	SC: 25–34 wt%; IRP: 1.3–1.6 mg L^−1^ (after 1 d)	HUVECs; SD rats	Cell morphology (D); MTT cellular activity (D); scratch migration (D); tube formation in Matrigel (I); tibiofibular fracture	1, 3 d; 1, 3 d; 2 d (1 d preculture); 16 h; 4 w	Nano‐gridding in combination with Sr promotes angiogenic behavior of HUVECs.	The addition of Sr to the nano‐gridded surface enhanced the adhesion, migration, and tube formation of HUVECs, and vascularization of newly formed bone.	^[^ ^132^ ^]^
Sr‐bioactive glass microspheres	6.227 mg L^−1^ (extract)	HUVECs; SD rats	Immunofluorescent staining (I); RT‐qPCR (I); calvarial defect	3 d; 3 d; 1, 6 w	The material contained Si which is a known direct proangiogenic stimulant.	SrBGM can enhance angiogenesis through regulation of an immune reaction.	^[^ ^128^ ^]^
SCPP	MC: 8 mol%; no IRP	HUVECs	MTT proliferation assay (D); cell morphology in SEM (D); ELISA (D); RT‐qPCR (D)	1, 3, 5, 7 d; 7 d; 7 d; 7 d	SCPP presented smoother surface than CPP; presence of Sr prevented formation of hydrogel.	SCPP resulted in higher proliferation rate, secretion of angiogenic genes, and better adhesion and spread of HUVECs.	^[^ ^127^ ^]^
Sr‐doped bioactive glass nanoparticles	MC: 8.5 mol%; No IRP	HUVECs	Alamar Blue cellular activity (D); cell distribution (D);	1, 3, 7 d; 1, 3, 7 d	Nanoparticles favored the spread and attachment of HUVECs.	Sr had a positive effect on the behavior of HUVECs.	^[^ ^121^ ^]^
Sr_5_(PO_4_)2SiO_4_	13–27 mg/ml (extract);	HUVECs	MTT proliferation (I,D); cell morphology (I); RT‐qPCR (I); cell attachment (D)	1, 3, 7 d; 1 d; 7 d; 1, 3, 7 d	Angiogenic Si was incorporated in the material. As a control, TCP was used.	The SPS scaffold enhanced angiogenic differentiation, attachment, and proliferation of HUVECs.	^[^ ^85^ ^]^
SCPP	MC: 8 mol%; IRP: 0.08 mg L^−1^ (after 7 d)	HUVECs (cc with OB); New Zealand white rabbits	MTT cellular activity (D); cell morphology (D); RT‐qPCR (D); ELISA (D); calvarial defect	1, 3, 7, 10, 14 d; 7 d; 7 d; 7 d; 8 w	SCPP presented a more compact surface in contrast to CPP and HA.	The SCPP scaffold promoted angiogenic behavior of both cell types in vitro and also in vivo in newly formed bone.	^[^ ^86^ ^]^
Sr‐doped bioactive glass	MC: 0.1 wt%; No IRP	Eahy926; Wistar rats	SulfoRhodamin B proliferation (I);femoral defect	1, 3, 6 d; 4, 7, 15, 30, 60 d	Incorporation of Sr into the BG decreased the oxidative stress thus contributing to bone repair.	Stimulated proliferation of ECs.	^[^ ^129^ ^]^

The effect of strontium ranelate (SrR), commercially known as Protelos/Protos, an antiosteoporotic drug, on ECs was discussed in ref. ^[^ ^124^ ^]^. The group reported a stimulatory capability of strontium on migration and tube formation properties of HUVECs with concentrations of around 7.5 mg L^−1^ Sr^2+^. However, they also addressed concerns about the increased incidence of cardiovascular events associated with the systemic use of SrR, which were also discussed in other studies.^[^ ^125^ ^]^ Nevertheless, they concluded that locally administered doses of the agent, smaller than those required by the oral intake of Protelos, should not be regarded as high risk.

Several publications discussed the effect of strontium incorporated in calcium‐polyphosphate scaffolds (CPP).^[^ ^86^, ^126^, ^127^ ^]^ Their findings, which complied with other strontium containing bioceramics,^[^ ^85^, ^121^, ^128^, ^129^, ^130^ ^]^ generally implied enhanced proliferative and migration capacity, and higher tube formation ability of ECs in the presence of strontium. Titanium‐based alloys with strontium incorporated on their surface yielded similar results.^[^ ^16^, ^131^, ^132^ ^]^ The studies reported improved adhesive, migration, and tube formation properties in vitro, suggesting likely enhanced vessel formation in vivo.

A group of researchers reported varying surface morphologies between CPP‐doped with strontium (SCPP) and without (CPP) and discussed their possible effect on ECs activity. The SCPP presented larger and more interconnected pores, resulting in a smoother surface with greater amounts of Ca^2+^ and (PO4)^3–^, and appeared to be favorable for ECs.^[^ ^86^, ^126^, ^127^ ^]^

The concentration of Sr^2+^ improving the angiogenic behavior from studies that included the ion release profiles ranged from less than 1.0 to several milligrams per liter. Most findings on optimal Sr^2+^ concentrations for ECs were within the range of 0.1–6.0 mg L^−1^ (cumulative release after 7 days or extract with constant concentration).^[^ ^16^, ^77^, ^86^, ^128^, ^130^ ^]^ Higher concentrations between 13 and 27 mg L^−1^, released from a bioceramic material, were reported by Zhu et al.^[^ ^85^ ^]^

### 5.5 Zinc

Another abundant trace element found in the human body is zinc. Zinc is important for many biological reactions and plays an essential role in the metabolic processes of bone. Next to magnesium, it is another biocompatible biodegradable metal, with its corrosion rate being somewhat lower than that of magnesium.^[^ ^70^ ^]^

Ten articles identified within the literature search are summarized in **Table** **7**. The findings show that Zn can be incorporated in coatings on metallic substrates^[^ ^69^, ^104^, ^107^, ^133^ ^]^ as well as in bioglass and other ceramic materials.^[^ ^14^, ^67^, ^68^ ^]^ Similar to magnesium and copper, the angiogenic ability of zinc is strongly dependent on its concentration and high doses can have adverse effects on the viability of ECs. An investigation of the effect of pure metal zinc on ECs showed that low concentrations of zinc of up to 3.92 mg L^−1^ (60 µm) promoted the angiogenic behavior of ECs, while higher doses inhibited their activity.^[^ ^70^ ^]^ This was in line with other observations of positive effects on ECs at concentrations of 1.4 mg L^−1^ after 7 days of culture,^[^ ^67^ ^]^ although even higher concentration of up to 32.5 mg L^−1^ favoring the migration activity of ECs was reported.^[^ ^104^ ^]^

**Table 7:** Literature overview of the effects of zinc on ECs

Zinc							
Tested material	Effective conc./ion release Zn^2+^	In vitro cell line/in vivo species	Assays—Direct (D)/indirect (I)	Incubation time	Other material properties	Results	Ref
Zn‐P coating on Zn	SC: 25 at%; 30 mg L^−1^ (extract)	EA.hy926	MTT viability (I); cell adhesion and morphology (D)	1, 3, 5 d; 3 d	‐	The ZnP coating improved the cytocompatibility of pure Zn and enhanced the attachment and viability of ECs.	^[^ ^133^ ^]^
PCL‐nHA‐nZnO	N/A	HUVECs (cc with OB); chicken embryos	MTT proliferation (D); cell morphology (D); migration into the scaffolds (D); RT‐qPCR (D); chick embryo chorioallantoic membrane assay	1–7 d; 3 d; 7 d; 7 d; 2 d	Secondary pores resulting from the surface modification with ZnO.	The in vivo assay in chicken embryo showed increased blood vessel formation in the presence of ZnO on the surface.	^[^ ^134^ ^]^
Silk fiber w. Zn + Si‐BrC brushite	MC: 0.25 wt%; No IRP	Porcine endothelial cells; New Zealand white rabbits	Tube formation in collagen (I); Alamar Blue proliferation (D); viability assay with PI (D); NO release (D); femur defect	N/A; 1, 3, 7 d; 7 d; 1, 7 d; 1, 3 month	‐	Positive synergistic effect of Si/Zn on angiogenesis.	^[^ ^68^ ^]^
Mg‐Zn‐Mn alloy	MC: 1 wt% Zn; No IRP	HUVECs	DNA synthesis capacity (BrdU) (I); MTT viability assay (I); tube formation in Matrigel (I); Western blot (I); RT‐qPCR (I)	24, 48 h; 24, 48, 72, 96, 120 h; 16 h; N/A; N/A	‐	The 6.25% Mg‐Zn‐Mn‐alloy extract could improve the angiogenic behavior of HUVECs, however no direct effect of Zn is discussed.	^[^ ^69^ ^]^
Cu/Zn‐calcium phosphate	MC: 1.3 g/l; IRP: 1.4 mg L^−1^ (after 7 d)	Vascular endothelial cells (cc with BMSCs)	Cell morphology (D); DAPI staining (D); CCK‐8 Proliferation assay (D); ELISA (VEGF) (D)	5 d; 5 d; 1, 4, 7 d; 14 d	Addition of dopant resulted in cubical nano‐/microparticles on the surface, depending on the concentration.	Cu/Zn co‐dopant system improved angiogenic capacity of HUVECs in cc with BMSCs.	^[^ ^67^ ^]^
ZnO‐polymer nanocomposite	MC: 0.8–1.6 wt%; No IRP	HUVECs; Wistar rats	Cell attachment evaluation in SEM (D); MTT cell viability assay (D); LDH assay (D); Subcutaneous implantation	24 h; 24 h; 24 h; 7, 21 d	‐	Scaffolds with of 1 and 2 wt% of ZnO resulted in better angiogenic behavior of HUVECs and blood vessel formation in vivo.	^[^ ^135^ ^]^
Zn/Mg‐titanium	IRP: ≈0.02 mg L^−1^ (after 7 d)	HUVECs	CCK‐8 Proliferation assay (D); RT‐qPCR (D); immunofluorescence analysis (D); intracellular zinc detection	1, 4, 7 d; 10 d; 10 d; 10 d	‐	Zn ions alone did not show significant improvement in angiogenesis, however, when combined with Mg, it has a positive effect.	^[^ ^107^ ^]^
Zn^2+^	3.9 mg L^−1^	HCECs (artery ECs)	MTT viability; BrdU proliferation; cell adhesion; centrifugation assay; cell spreading; scratch migration; cell morphology; RT‐qPCR	24 h; 24 h; 2, 6 h; 2, 6 h; 0, 2, 4, 6, 8 h; 0, 6 h; 24 h; 24 h		Low concentration of Zn (up to 60 µm = 3.9 mg L^−1^) promoted angiogenic behavior of HUVECs.	^[^ ^70^ ^]^
Bioactive glasses with Zn	N/A	N/A	N/A review	N/A	‐	Zn in certain concentration promoted angiogenic behavior of HUVECs.	^[^ ^14^ ^]^
Ca‐P‐Zn‐Cu coating on Ti	6.5–32.5 mg L^−1^ (supplemented media)	HUVECs	Tube formation in collagen gels; transwell migration	24, 48 h; 4 h		Improved migration ability with Zn of up to 32.5 mg L^−1^.	^[^ ^104^ ^]^

Direct (D) assay = culturing cells directly on the material's surface; Indirect (I) assay = culturing cells with the material's extract; Abbreviations: MC = material composition; SC = surface composition; IRP = ion release profile; d = days; w = weeks; cc = coculture; ECs = endothelial cells; OB = osteoblasts.

Owing to its high corrosion rate, supplementary surface modification was often adopted to control the ion release and improve the cytocompatibility of zinc.^[^ ^133^ ^]^ Zinc was often found as a co‐doped agent in combination with other bioactive elements such as copper,^[^ ^67^ ^]^ magnesium,^[^ ^69^, ^107^ ^]^ phosphorus/calcium,^[^ ^67^, ^133^, ^134^ ^]^ or silicon,^[^ ^14^, ^68^ ^]^ which also reported improved viability of ECs in vitro^[^ ^14^, ^135^ ^]^ and formation of blood vessels in vivo.^[^ ^134^ ^]^ However, the positive effects were often attributed to the synergistic effect of zinc and the other element.

## 6 Discussion

Aseptic loosening is recognized as one of the leading causes of implant failure after primary THA. Through ion and particle doping, angiogenesis and osteogenesis boosting agents can be introduced onto the surfaces of bioinert biomaterials (such as titanium) and thereby strengthen the attachment at the interface and very likely improve the failure odds. The results summarized in the previous section (Section 5) show that all reviewed elements (copper, magnesium, silicon, strontium, and zinc) present a concentration‐dependent angiogenic potential. In this section, the properties of these elements will be further discussed with respect to the methodological approach used for angiogenic assessment. Furthermore, the elements will be compared based on their angiogenic mechanism of action and their effect on other cell types engaged in the bone repair process. Finally, the acquired knowledge will be utilized to propose a solution, which could improve osseointegration of a permanent implant through the effective delivery of dual angiogenic and osteogenic promoters from the biomaterial's surface.

### 6.1 Assessment Methods for Angiogenesis Induced by Inorganic Agents

For this review, publications discussing the interaction of one or multiple of the selected elements with ECs are compared. Direct (D) and indirect (I) testing strategies of the materials were identified; the cells were either seeded directly on the surface or cultured with extracts (also referred to as degradation fluid or conditioned medium) of the respective biomaterial on a standard culture dish. Depending on the biomaterial, the two approaches can yield different results and their mutual comparison may not be accurate.^[^ ^14^ ^]^ Extracts obtained from the material stimulate the cell only via released ions/particles (chemical composition) from the biomaterials. On the other hand, the cells in direct contact with the biomaterial will be also affected by its surface morphology, wettability, or surface energy.^[^ ^138^, ^139^, ^140^, ^141^ ^]^ Several studies have discussed the morphological changes of the biomaterial's surface and its potential effect on the ECs. For example, the changes of the surface of the biomaterial achieved by the incorporation of silicon were reported to be substantial for attachment, spreading, and further activity of cells.^[^ ^92^, ^111^ ^]^ However, they did not provide both sets of data (from direct and indirect testing of the biomaterial), which could clarify the hierarchy of the chemical and physical stimuli, that is, which one is primary for initiation of the desired cellular response. Generally, treating cells with extracts is relatively simple while seeding, detaching, and collecting cells from a biomaterial with complex surface morphology require optimized protocols which are methodologically more challenging and laborious.

The vast majority of biomaterials found in this review were very complex with multiple (bioactive) elements in their composition. Despite the rigorous testing of different concentrations of the studied elements, the other bioactive agents present in the biomaterials and the possible additive/synergistic effect must always be taken into consideration and ideally should be compared with studies examining the effect of pure ions^[^ ^70^, ^78^ ^]^ or very simple molecules separately.^[^ ^124^ ^]^

The incubation time of the ECs with the various biomaterials/ions varied per study, but was generally in the range of several days (some studies reported incubation of only a few hours). This experimental variable also affects the results as short incubations might not be sufficient to take any/the full effect and elicit response whereas long incubations may lead to undesired effects as well, such as toxicity.

Focusing on methodologies, the most frequently studied cell properties related to angiogenesis are proliferation, migration, and sprouting. Due to their simple protocol, colorimetric assays are often chosen for the determination of cellular proliferative activity. For the ambiguous character of the output data and their potential misinterpretation,^[^ ^72^, ^142^ ^]^ the number of studies included in this review which interpreted the results of colorimetric metabolic assays as proliferation data rather than metabolic activity was concerning. The highly reducing environment does not necessarily reflect the higher number of cells as this may also be the result of increased metabolic activity due to stimulating biochemical cues. Therefore, careful data interpretation is essential and the use of another assay (such as DAPI cell count) for validation of the obtained results is strongly recommended.^[^ ^53^, ^54^, ^72^ ^]^ An obstacle that can be encountered using DAPI and other fluorescent imaging methods is autofluorescence of certain materials (such as some polymers).^[^ ^143^ ^]^

A weakness of the scratch/wound healing assay, assessing the cell motility, is its reproducibility, as the size of the scratch is not always uniform. Additionally, it should be noted that the wound closure is not necessarily accomplished by migration alone, and the contribution of proliferation should be considered as well.^[^ ^53^, ^54^, ^57^, ^72^ ^]^ Finally, the transwell assay allows for testing with extracts or conditioned media only, while the wound healing assay can be performed also on substrates with smooth surfaces allowing to create a scratch in the cellular monolayer.

The tube formation assays were mostly performed in Matrigel‐coated wells. Despite its relatively high price, its batch‐to‐batch composition variation, and the fact that it is derived from murine breast tumor tissue, it seems to be the standard material for this assay. However, its high growth factor content has been demonstrated to induce an atypical tendency toward the formation of tubular structures by non‐ECs.

Phenotype commitment is most frequently assessed using RT‐qPCR and ELISA methods (quantification of VEGF, HIF1‐*α*, and PECAM1 expression) and detection of released NO ions. Besides the already discussed angiogenesis‐related growth factors and molecules (HIF1‐*α*, VEGF, and PECAM1), the process is also guided by a number of other signaling pathways involved in transcriptional and post‐translational regulation. Wnt pathways are groups of signaling proteins mediating cellular proliferation, migration, differentiation, survival, and apoptosis, and they are potent guides for bone healing and vessel remodeling. With regards to angiogenesis, Wnt/ß‐catenin is one of the known Wnt pathways governing the transcription of genes associated with vascular growth (VEGF).^[^ ^144^, ^145^, ^146^, ^147^ ^]^ Notch signaling ligands and receptors are involved in vascular homeostasis,^[^ ^148^, ^149^, ^150^ ^]^ regulating phenotype commitment of endothelial tip and stalk cells responsible for migration and proliferation, respectively, during vascular sprouting.^[^ ^148^ ^]^

None of the studies included in this review investigated the effect of ions on the first stage of angiogenesis, which is the basal membrane degradation. A possible explanation could be that researchers consider an already broken/damaged basement membrane in their models and do not feel the need to address it. Those assays may, however, be highly relevant for assessment of osteoconductive scaffolds supporting large defects/injuries and requiring regeneration of greater portion of bone and its vasculature.

Studies testing the response of cells seeded directly on the biomaterials often included observation of the cellular morphology. This simple experiment grants direct (although not quantitative) feedback about the biomaterial cytocompatibility for ECs.

All in all, there is a wide spectrum of available methods for assessment of angiogenic behavior of ECs in 2D. The recommended approach drawn from the findings of this review is in favor of testing multiple behavioral features of ECs in the presence of a potential angiogenic stimulus in order to evaluate its angiogenic potential. Moreover, it is advisable to perform cell cultures in extracts obtained from the biomaterials (indirect test), and on biomaterial's surfaces (direct test) to decouple and distinguish between effects of chemical composition and physical properties of the used biomaterial, as they both play a significant role in the cellular response. Considering the growing trend of porous and degradable biomaterials necessitating proper bone ingrowth, relevant angiogenic models with transition from 2D to 3D will become a fundamental aspect of research dealing with osteoconductive biomaterials. The 3D methodologies lay ground for closer approximation of intercellular interactions and their innate matrices which as well are pivotal for tissue regeneration.^[^ ^146^, ^151^ ^]^

### 6.2 Role of Ions in Angiogenesis at the Implant‐Bone Interface

An ideal element should feature a dual incentive toward angiogenic and osteogenic commitment of ECs and MSCs/osteoblasts, respectively, and thereby simultaneously promote blood vessel and bone matrix formation. All reviewed elements (copper, magnesium, silicon, strontium, and zinc) demonstrated pro‐angiogenic characteristics at certain concentrations (discussed in Section 5). Considering the relatively wide range of effective concentrations reported in the different studies, the obtained responses of ECs to the elements were very likely conditioned by additional factors, such as other released ions from the biomaterials or physical properties of the substrates.

The role of zinc in blood vessel formation has been ascribed to its regulatory actions toward VEGF secretion through its high affinity to zinc proteins,^[^ ^152^ ^]^ and other zinc sensing receptors,^[^ ^153^ ^]^ which can additionally promote survival and growth of ECs through activation of intracellular signaling pathways. The enhancing effects of magnesium on migratory properties of ECs, on the other hand, have been associated with its chemoattractant role^[^ ^154^ ^]^ and increased integrin function.^[^ ^155^ ^]^ Both magnesium and zinc show auspicious potential for scaffolds in bone tissue engineering applications for their biodegradable properties. However, according to the current research, their degradation process has not been well contained yet. This may be reflected by the concentrations of released magnesium and zinc ions, which were usually much higher than concentrations detected with copper, silicon, or strontium. The high corrosion rate of both reviewed metals (magnesium and zinc) can result in adverse effects on bone regeneration: in the case of magnesium scaffolds, uncontrollable development of hydrogen bubbles and alkaline environment have been shown to severely inhibit the osteogenic process.^[^ ^13^ ^]^ In addition, such a scaffold might not ensure the required mechanical stability throughout the healing process until the new bone tissue is formed.^[^ ^136^ ^]^ The current attempts to moderate the negative effects of the rapid corrosion include alloying with other more stable metals or surface modifications, possibly making the fabrication process excessively complex. Incorporation of Mg on the surface of a permanent orthopedic implant needs further scrutiny regarding surface design, taking into account the effects on both the osteogenic and angiogenic processes.^[^ ^75^, ^106^ ^]^

The clear superiority of copper for bone tissue engineering and vascular applications is attributed to its dual antibacterial and angiogenic capacity. Even in relatively small concentrations, copper can mitigate the risks of fatal peri‐implant bacterial infection, leading to septic loosening of a prosthesis.^[^ ^13^, ^87^, ^101^ ^]^ Besides its antibacterial activity, copper could potentially accelerate bone healing through enhanced angiogenesis. The mechanism by which copper promotes the formation of new blood vessels is based on the stabilization of HIF1‐*α* and further stimulation of VEGF expression.^[^ ^13^, ^36^, ^91^ ^]^ Despite the inherent role of copper in the bone metabolic processes, several publications reported severe sensitivity and possible inhibitory effects of copper on MSCs and osteoblasts at concentrations which, at the same time, were found to be beneficial for ECs.^[^ ^78^, ^156^ ^]^ Regarding orthopedic applications, an appropriate amount of copper favoring both cell types must be carefully chosen to avoid compromising the bone healing process. Alternatively, fabricating a coating with a properly tuned ion release profile could systematically stimulate the most relevant cells in the individual stages of the healing process and thereby effectively promote bone regeneration.

Another element with a dual character is silicon. Owing to its favorable properties for endothelial and osteoblastic cells, which have been known for years, it is being employed for applications in tissue engineering, including solutions for bone regeneration, where positive interactions with both cell types are crucial.^[^ ^65^, ^111^, ^115^ ^]^ Silicon is a stable element and unlike copper, magnesium, or zinc, it does not exhibit as many risks regarding possible cytotoxicity and it is the only element in this study that is well accepted by tissues even in large concentrations (it is present in the majority of active bioceramic materials). Its mechanism of action is analogous to that of copper: it increases expression of proangiogenic molecules, such as VEGF and FGF, it activates kinase insert domain receptor and stimulates the production of NO.^[^ ^157^, ^158^ ^]^

Strontium is currently known for its excellent capacity to encourage the formation of new bone and represents a new generation of promising orthopedic solutions.^[^ ^27^, ^30^ ^]^ Due to its mechanism of action, it can promote bone formation more effectively than calcium, and most likely, it also surpasses the capacity of silicon to secure a strong attachment with the implant. Despite the intensive research, little has been reported regarding its effect on ECs. The data from the reviewed literature indicate that strontium can favor the viability of ECs and also promote angiogenesis by stimulating MSCs to produce VEGF.^[^ ^124^, ^127^ ^]^ The exact mechanism by which strontium activates ECs and guides their angiogenic behavior is not yet fully known, however, the involvement of the calcium‐sensing receptor (CaSR) has been discussed.^[^ ^159^ ^]^ This receptor is inherently involved in the mechanism of strontium‐facilitated osteogenesis. It can bind strontium instead of calcium due to its similar atomic and ionic properties.^[^ ^28^, ^123^ ^]^ Confirming the role of CaSR in the strontium‐mediated angiogenic commitment of ECs would introduce a new and possibly very effective system for the early development of well‐vascularized bone.

Taken together, the comparison of the five elements and requirements for the intended application (**Table** **8**) suggests that strontium and silicon could be a superior choice to the other three elements with the currently available processing methods. The aims to utilize zinc and magnesium are challenged by their rapid corrosion (improperly controlled ion release could result in adverse effects), while copper may hamper the osteogenic process at concentrations beneficial for ECs. Strontium is known for its tremendous potential to promote osteogenesis, for which it has been utilized in osteoporotic treatments in the past, and the findings of this study imply promising results for the endothelial interaction as well. Silicon is utilized across many tissue engineering areas and presents a dual angiogenic and osteogenic activity.

**Table 8:** Overview of the criteria assessment per element

	Intended use	Tolerable ion content for ECs	Angiogenic properties	Osteogenic properties	Other properties	Current biocompatibility for bone applications
Cu	Cardio. and ortho. applications	20 mg L^−1^	Excellent	Inhibitory effect at high conc.	Antibacterial properties	Good
Mg	Degradable scaffolds	190 mg L^−1^	Good	Good	Biodegradability	Low due to high corrosion rate and cytotoxicity due to degradation products
Si	All tissue engineering	27 mg L^−1^	Good	Good	Applicability for various tissues	Excellent
Sr	Ortho. applications	27 mg L^−1^	Good	Excellent	Antiosteoporotic drug	Good
Zn	Degradable ortho. scaffolds	32 mg L^−1^	Good	Good	Biodegradability	Low due to high corrosion rate (the same consideration as for Mg)

### 6.3 Angiogenic Response of ECs Mediated through Other Cell Types

Indirect cellular interactions with materials, mediated via other cell types, are certainly of great importance as they represent a closer approximation of the in vivo situation in in vitro models. Although they were not the main focus of this review, some included publications^[^ ^16^, ^67^, ^86^, ^126^, ^131^ ^]^ discussed these interactions and therefore will be briefly addressed in this section.

The mutual interaction of MSCs, osteoblasts, chondrocytes, fibroblasts, and immune cells with ECs is certainly vital for proper fracture repair. Although a coculture of MSCs/osteoblasts with ECs is the most commonly used model for bone fracture‐related angiogenesis, these cells do not interact until later in the healing process. The initial inflammatory reaction, with the onset of angiogenesis, is guided by immune cells. The description of the relationship between macrophages and ECs showed an improved angiogenic response of ECs cultured in conditioned medium from stimulated mouse monocytes.^[^ ^128^ ^]^

ECs thrive in the presence of MSCs/osteoblasts and vice versa. The angiogenic and osteogenic differentiation potentials are higher in comparison to respective monocultures, leading to successful bone regeneration.^[^ ^96^, ^97^ ^]^ MSCs belong to a group of cell types capable of VEGF secretion. Via paracrine signaling pathways, this cytokine can mediate the activity of ECs, including their differentiation, proliferation, and migration.^[^ ^16^, ^94^, ^160^ ^]^ The performed experiments showed that cocultures of ECs and MSCs were beneficial for differentiation of endothelial phenotype and expression of specific markers, such as CD31 and von Willebrand factor, likely due to the delivery of VEGF to ECs.^[^ ^94^ ^]^ The symbiotic relationship of the coculture has also been illustrated by the mutual attachment of MSCs and ECs (particularly EPCs), which augments the pluripotency of MSCs and simultaneously promotes angiogenesis.^[^ ^95^, ^161^ ^]^

Under optimal conditions, VEGF production by MSCs can be increased. The use of strontium‐containing titanium material was reported to stimulate MSCs toward higher secretion of VEGF and platelet‐derived growth factor‐BB, which are both essential for angiogenesis.^[^ ^16^ ^]^ In their experiments, much higher concentrations of those molecules were detected in conditioned medium obtained from MSCs cultured with strontium, which subsequently ensured greater recruitment and tube formation capacity of HUVECs. An experiment yielding similar findings was described in other studies, using conditioned media from MSCs stimulated by Sr^[^ ^131^ ^]^ and Mg^[^ ^76^ ^]^ ions, respectively and, for EC cultures.

These findings support the arguments, that despite their importance in the initial stages of research, monocultures are not an optimal representation of the in vivo interactions, and they further imply that the angiogenic function should be assessed from a broader angle. Generally, it also suggests that elements, which do not necessarily trigger ECs could still (strongly) boost the blood vessel formation indirectly through stimulation of other cell types and subsequent activation of ECs. The relationship between MSCs and ECs, which has proven to serve as an example, is critical for angiogenesis and most likely determines the outcome.

### 6.4 Future Perspectives

Considering the causes leading to failures of permanent hip implants, promoting bone formation, and strengthening the attachment at the interface could potentially reduce their aseptic loosening, which is usually attributed to the insufficient bioactivity of those biomaterials.

The fracture healing model is used to emulate the bone repair process after replacement surgeries. This complex set of events, which is governed by many molecular cascades and environmental factors, can be modulated by various physical or biochemical agents interfering in individual of multiple stages of this process. The scientific evidence for the mutual dependency between bone matrix deposition and blood vessel formation, and its role in the fracture healing process, has commenced the development of biomaterials, which could promote both processes simultaneously through relevant agents, and thereby ensure early deposition of well‐vascularized bone and secure a stronger connection with the implant.^[^ ^15^, ^22^ ^]^

The current strategies to improve angiogenesis usually rely on the favorable environment created by structures with pores of appropriate volumetric ratio, which allow for vessel ingrowth.^[^ ^11^, ^18^ ^]^ Those, however, must ensure complete interconnectivity, else they hinder the cellular invasion and formation of a new vascular network.^[^ ^162^ ^]^ Local delivery of proangiogenic factors such as VEGF is limited by natural properties of those molecules, including low protein stability and short circulating half‐life and therefore their therapeutic use compels advanced engineering methods.^[^ ^22^, ^163^, ^164^, ^165^ ^]^ Angiogenic stimulation through inorganic ions^[^ ^25^ ^]^ offers another approach with a potentially tunable release profile of the active element adapted to the needs of the different stages in the healing process.

In this review, five inorganic elements (copper, magnesium, silicon, strontium, and zinc) were analyzed and compared with respect to their angiogenic capacity. Taking into account the currently available surface biofunctionalization methods, the properties of silicon and strontium showed the best match with the defined criteria. Both elements present low or no risk of cytotoxicity, effectively promote osteogenesis, and this review confirmed also their angiogenic potential. Therefore, a suitable approach would be to design titanium‐based implants with silicon and/or strontium‐doped surfaces, which could deliver angiogenic and osteogenic stimuli simultaneously and in a controlled manner.

The incorporation of such agents can be achieved through various processes, for example, chemical and physical vapor deposition, electrochemical deposition, or plasma spraying.^[^ ^6^, ^166^ ^]^ Electrochemical methods are often preferred for their relatively short procedure, applicability for large and complex titanium substrates, and a wide range of elements/molecules, which can be incorporated on the surface.^[^ ^167^, ^168^ ^]^ Moreover, by altering the input parameters, such as time, applied potential, and electrolyte composition, the methods can produce a surface with desired (tailored) topography. One of the available electrochemical methods is PEO (also known as micro‐arc oxidation), which generates a porous oxide layer through local plasma discharges.^[^ ^169^, ^170^ ^]^ Silicon and strontium can be both incorporated into the surface of titanium‐based alloys through the PEO process^[^ ^75^, ^171^, ^172^ ^]^ and a gradual release of ionic products from the formed layer may lead to desired angiogenic and osteogenic effects at the affected site. Such a bioactive system should be first tested in vitro using the most relevant and accurate assays, as presented in this review. In addition, the cellular response to such biomaterials should be tested not only in monocultures of ECs, but also in cocultures of ECs and MSCs/osteoblastic cells to approximate the biomolecular interactions occurring during the mutually dependent processes, namely the vessel and bone formation. Only with a rigorous set of in vitro experiments as described above, followed by relevant in vivo studies, will any given biomaterial containing Cu, Mg, Si, Sr, or Zn prove itself as a superior implant in THA.

## 7 Conclusions

The role of angiogenesis in the fixation of permanent orthopedic implants in bone tissue has remained underinvestigated. Therefore, we have conducted a review of the angiogenic properties of trace elements (Cu, Mg, Si, Sr, and Zn) incorporated in the biomaterials’ surfaces. We have evaluated the assays used to study the response of ECs to these surfaces, made a comparative analysis of the angiogenic properties of the elements investigated, and evidenced the mechanism underlying their angiogenic properties.

The results described in this review showed that the methodological approach for angiogenic assessment comprised of similar in vitro 2D assays among the reviewed studies. Differences were identified in the incubation period of cells with the bioactive agent(s). The most frequently used assays included proliferation, migration, and sprouting assays followed by gene expression methods. All five reviewed elements (Cu, Mg, Si, Sr, and Zn) displayed in vitro pro‐angiogenic capacity, but were in some cases strongly concentration‐dependent. Silicon and strontium appear to be superior for orthopedic implants as agents with dual angiogenic and osteogenic properties, considering the currently available processing containment of those materials. They are known for their robust potential to promote osteogenic capacity and the findings in this study suggest promising results for the early development of vascularized bone.

## Conflict of Interest

The authors declare no conflict of interest.

## Supporting information

Supporting Information
