# Supplementary material for: Inorganic Agents for Enhanced Angiogenesis of Orthopedic Biomaterials
Source: Adv Healthc Mater. 2021 May 26;10(12):2002254. doi: 10.1002/adhm.202002254 (PMC11469191; doi:10.1002/adhm.202002254)
Supplement: Supplementary file 1 — Supporting Information [file ADHM-10-2002254-s001.pdf]

© 2021 Wiley-VCH GmbH

ADVANCED HEALTHCARE MATERIALS

Supporting Information

for *Adv. Healthcare Mater.*, DOI: 10.1002/adhm.202002254

Inorganic agents for enhanced angiogenesis of orthopaedic biomaterials

M. Šalandová1, I.A.J. van Hengel1, I. Apachitei1, A.A. Zadpoor1, B.C.J. van der Eerden2#, L.E. Fratila-Apachitei1*#*

Supplementary figures

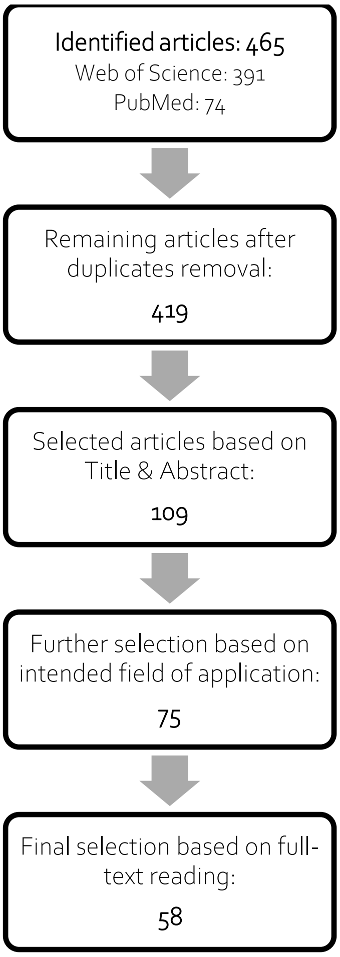

Figure S1. Flowchart presenting the literature screening process.
